# Complexity of the *Ruminococcus flavefaciens* FD-1 cellulosome reflects an expansion of family-related protein-protein interactions

**DOI:** 10.1038/srep42355

**Published:** 2017-02-10

**Authors:** Vered Israeli-Ruimy, Pedro Bule, Sadanari Jindou, Bareket Dassa, Sarah Moraïs, Ilya Borovok, Yoav Barak, Michal Slutzki, Yuval Hamberg, Vânia Cardoso, Victor D. Alves, Shabir Najmudin, Bryan A. White, Harry J. Flint, Harry J. Gilbert, Raphael Lamed, Carlos M. G. A. Fontes, Edward A. Bayer

**Affiliations:** 1 Department of Biomolecular Sciences, The Weizmann Institute of Science, Rehovot, Israel; 2 CIISA – Faculdade de Medicina Veterinária, Universidade de Lisboa, Avenida da Universidade Técnica, 1300-477 Lisboa, Portugal; 3 Department of Molecular Microbiology and Biotechnology, Tel Aviv University, Ramat Aviv, Israel; 4 Chemical Research Support, The Weizmann Institute of Science, Rehovot, Israel; 5 Department of Animal Sciences, Institute for Genomic Biology, University of Illinois at Urbana–Champaign, Champaign, IL, USA; 6 Department of Animal Sciences, University of Illinois at Urbana–Champaign, Champaign, IL, USA; 7 Microbiology Group, Rowett Institute of Nutrition and Health, University of Aberdeen, Foresterhill, Aberdeen, Scotland, UK; 8 Institute for Cell and Molecular Biosciences, Newcastle University, The Medical School, Newcastle upon Tyne NE2 4HH, UK

## Abstract

Protein-protein interactions play a vital role in cellular processes as exemplified by assembly of the intricate multi-enzyme cellulosome complex. Cellulosomes are assembled by selective high-affinity binding of enzyme-borne dockerin modules to repeated cohesin modules of structural proteins termed scaffoldins. Recent sequencing of the fiber-degrading *Ruminococcus flavefaciens* FD-1 genome revealed a particularly elaborate cellulosome system. In total, 223 dockerin-bearing ORFs potentially involved in cellulosome assembly and a variety of multi-modular scaffoldins were identified, and the dockerins were classified into six major groups. Here, extensive screening employing three complementary medium- to high-throughput platforms was used to characterize the different cohesin-dockerin specificities. The platforms included (i) cellulose-coated microarray assay, (ii) enzyme-linked immunosorbent assay (ELISA) and (iii) *in-vivo* co-expression and screening in *Escherichia coli*. The data revealed a collection of unique cohesin-dockerin interactions and support the functional relevance of dockerin classification into groups. In contrast to observations reported previously, a dual-binding mode is involved in cellulosome cell-surface attachment, whereas single-binding interactions operate for cellulosome integration of enzymes. This sui generis cellulosome model enhances our understanding of the mechanisms governing the remarkable ability of *R. flavefaciens* to degrade carbohydrates in the bovine rumen and provides a basis for constructing efficient nano-machines applied to biological processes.

Cellulose degradation has long been focus of many studies in the fields of renewable energy and waste management^1^,^2^,^3^,^4^,^5^. Cellulose is the most abundant naturally occurring organic material, yet its recalcitrant nature renders it largely unavailable for extensive biodegradation^6^,^7^. Herbivores feed on plants as a sole carbon source. The rumen is a highly populated and competitive ecological niche, where a complex and diversified repertoire of microbial enzymatic systems participate in deconstruction of recalcitrant carbohydrates through molecular mechanisms which remain poorly understood^8^,^9^,^10^. An enormous concentration of archaea, protozoa, fungi and bacteria colonize the rumen. Although only a small fraction of these microbes are directly engaged in fiber degradation, they all benefit from the metabolic by-products. Dominant rumen species identified as primary degraders of crystalline forms of polysaccharides are fibrolytic bacteria, namely *Fibrobacter succinogenes, Ruminococcus flavefaciens* and *Ruminococcus albus*^9^,^11^.

*R. flavefaciens* is a Gram-positive, anaerobic bacterium of the *Firmicutes* phylum. It is the only known bacterium in the rumen shown to possess a definitive cellulosome, *i.e.*, a discrete multi-enzyme complex specialized in the breakdown of cellulose and associated plant cell-wall polysaccharides^12^,^13^,^14^. The cellulosome complex carries three fundamental features. Firstly, cellulosome assembly results from the incorporation of cellulosomal enzymes, e.g. glycoside hydrolases (GH), carbohydrate esterases (CE), and polysaccharide lyases (PL), into structural scaffoldin subunits through high-affinity interactions between cohesin and dockerin modules. Cohesins are modular components of scaffoldins, whereas dockerins are borne by individual cellulosomal enzymes that are integrated into the complex through interaction with the cohesins^15^,^16^,^17^,^18^. Secondly, cellulosomes are anchored to the cell-surface through a mechanism, which may take place either covalently through enzymatic mediation or non-covalently via a specialized module^19^,^20^,^21^. Thirdly, a non-catalytic substrate (carbohydrate)-binding module (CBM) attaches the entire complex to cellulose^22^,^23^,^24^. Cellulosomes thus present a complex functional machinery of great environmental flexibility and adaptation, gained by the many possible arrangements of its modular components, as dictated by the deployment of different cohesin-dockerin pairs.

The profile of *R. flavefaciens* presents a multiplicity of rumen strains, both similar to and phylogenetically distinct from previously discovered strains^25^,^26^,^27^. All members of this species have been shown to possess a scaffoldin-encoding *sca* gene cluster, and thus appear to synthesize a cellulosome. The locus encodes scaffoldins ScaC, ScaA, ScaB and ScaE, as well as a CttA protein, believed to include two consecutive carbohydrate-binding modules (CBMs)^26^. *R. flavefaciens* strains have in common an enzyme-integrating subunit, ScaB, which carries a C-terminal X module-dockerin (XDoc) dyad that in turn recognizes the single cohesin of the surface-anchored scaffoldin, ScaE^28^,^29^. ScaE is covalently linked to the bacterial envelope via an LPXTG motif, mediated by the enzyme sortase; thus the entire multi-enzymatic cellulosome assembly is bound to the bacterial cell surface^21^. In addition, the ScaE cohesin also binds the CttA protein, which, like ScaB, carries a C-terminal XDoc dyad and would thus promote substrate targeting and bacterial adhesion via its CBM modules, thereby initiating deconstruction of the cellulosic substrate. Moreover, the XDoc modules of CttA and ScaB include three unique insertions within their structure, recently proposed to mechanically support the bulky complex and its anchoring to the cell via ScaE^23^,^30^,^31^.

The main difference among the various *R. flavefaciens* strains is the number and types of cohesins borne by the main ScaB subunit and their specificity(ies) towards cognate dockerins. In strain FD-1, ScaB harbors nine cohesins, four of which (cohesins 1–4) are similar in sequence to the two ScaA cohesins, whereas the others (cohesins 5–9) bind to the unique ScaA dockerin. Previous studies have demonstrated variation in scaffoldin recognition by different classes of enzymes in *R. flavefaciens.* Some enzymes bind directly to ScaA and ScaA-like cohesins on ScaB, whereas others bind via the intermediary ScaC cohesin^32^, which acts as a selective “adaptor” scaffoldin that alters enzymatic composition of the cellulosome. These divergent interactions and their significance towards cellulosome organization are presumably governed by the sequence and consequent specificity of the enzyme-borne dockerin.

In the past, cohesins were distinguished into three types: I, II and III, based on phylogeny of the primary sequences. Likewise dockerins that interacted with these cohesins were regarded as the same type. The cohesins and dockerins of *R. flavefaciens*, belong to type III albeit with considerable internal diversity (Fig. 1). Curiously, the ScaC cohesin of *R. flavefaciens* maps onto a divergent phylogenetic branch, closer to those of the clostridial type-I cohesins (Fig. 1). Only a single enzyme-borne dockerin, CE3B, a family 3 carbohydrate esterase, had been shown previously to bind to the ScaC cohesin, whereas the general binding specificity and range of proteins it serves to integrate remains obscure^28^.

A draft genome of *R. flavefaciens* strain FD-1 has been published, revealing 223 dockerin-containing ORFs^29^,^33^. This is triple the number of cellulosomal components observed for clostridial species, rendering the *R. flavefaciens* cellulosome the most intricate described to date. The bacterium comprises an abundant repertoire of catalytic and CBM modules frequently organized in multi-modular protein architectures^34^. The presence of numerous genes encoding for highly complex multi-modular hemicellulases is particularly striking. Nevertheless, many of the dockerin-bearing parent proteins appear to be unrelated to traditional cellulosome activities, with predicted functions, such as serpins, peptidases, LRR (leucine-rich repeats) proteins and transglutaminases.

The dockerin sequences of *R. flavefaciens* FD-1 exhibit great sequence diversity that ranges between 20–98% homology. This has led to their categorization into six distinct major groups and eleven sub-groups, based on sequence conservation patterns, secondary structural elements and postulated Ca^+2^-binding and cohesin-recognition residues^33^. Each group exhibits unique and recognizable features, such as the presence of an atypical number of conserved residues in the second repeat. Some dockerins resemble known dockerins (groups 3 and 6) and some are exclusive to *R. flavefaciens* FD-1 (groups 1–2). The conservation pattern of the group classification of the *R. flavefaciens* dockerins from Rincon *et al*.^33^ is available in Supplementary Figure S1.

Nonetheless, the functional significance of dockerin classification into these different groups remains unclear. It was thus uncertain whether the dockerin grouping reflected variation in ligand (cohesin) specificity or stability factors within the context of their parent proteins. To clarify these issues, the present report describes a combined experimental approach to investigate cellulosome configuration in *R. flavefaciens* strain FD-1.

## Results

### Selection of representative cohesin and dockerin modules

Past studies have predicted 223 genomically encoded dockerin-bearing proteins in *R. flavefaciens*^33^. Taken together with the 29 predicted cohesin modules^29^, a theoretical matrix of 6467 potential cohesin-dockerin interactions was generated. In this work, we accumulated data using three complementary experimental platforms to identify interacting cohesin and dockerin pairs that may shape cellulosomal architecture and enzyme composition in *R. flavefaciens* FD-1. Dockerin modules were selected to represent the previously established bioinformatic sequence diversity. Table 1 provides a list of the 77 dockerins selected for recombinant production and subsequent testing within the different experimental platforms. The selected dockerins originated from all of the different groups and subgroups^33^ as designated in the Table. The nature of the parent protein was also considered in dockerin selection. Thus, some dockerins belong to proteins bearing typical plant cell wall-degrading catalytic modules (e.g., various GH and CE families) while others are part of proteins containing structural or functional components (e.g., CBM, predicted cohesin-bearing scaffoldins, serpins and LRR motifs). In addition, dockerins belonging to proteins whose expression was upregulated by growth on cellulose were also targeted^34^ (e.g., Doc 11–13, Table 1). While most dockerins are located at the C-terminus of their host protein, a few are at the N-terminus or in the middle of the polypeptide chain (e.g., Doc 11–13, 36, 50 and 55, Table 1). The dockerin of the family 48 GH was also included (Doc 14, Table 1), since this enzyme represents a major contributing component of every cellulosome system thus far described.

**Figure 1:**
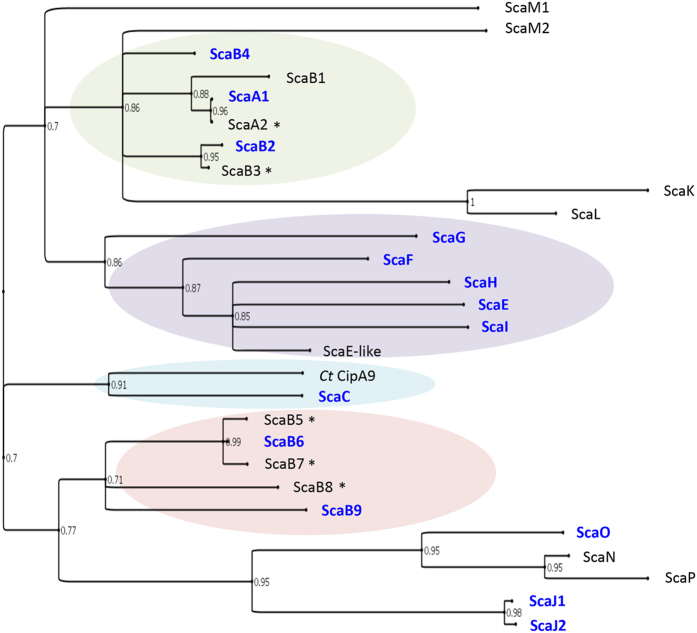
Phylogenetic tree of the *R. flavefaciens* FD-1 cohesins. Cohesins B1–B4 are located together in the tree (mint green), consistent with reports in the literature, i.e., closer to one another and to ScaA cohesins than to cohesins B5–9 (pink). Cohesins selected for the microarrays assay are shown in blue font. *C. thermocellum* CipA cohesin 9 (*Ct*CipA9) was used as a marker to represent type I cohesins. Note that the cohesin borne by the ScaC adaptor scaffoldin is associated with the type I cohesins (powder blue) and thus diverges from the type III *R. flavefaciens* cohesins. Another cluster of cohesins is marked in lavender. Asterisks (*) indicate cohesins tested in both complementary ELISA and non-denaturing PAGE studies. The tree was generated using PhyML software (http://www.atgc-montpellier.fr/phyml) and processed using FigTree v1.4.2 (http://tree.bio.ed.ac.uk/software/figtree). Bootstrap threshold of 0.7 is presented.

A collection of 19 cohesin modules was selected from the eight previously identified scaffoldins of the bacterium, including ScaA cohesins 1 and 2 (ScaA1–2), ScaB cohesins 2 to 9 (ScaB2–9), and the single cohesins in ScaC, ScaE, ScaF, ScaG, ScaH and ScaI (based on bioinformatic data, cohesins ScaB1–4 are highly similar;^29^ cohesins B2–B4 were thus selected and included as representatives but cohesin B1 was not included). Additionally, three putative cohesin modules were selected: ScaJ cohesins 1–2 (ScaJ1–2) and ScaO, whose sequence diverge from canonical cohesins (Fig. 1). The sequences of 19 selected cohesins are typical of type-III cohesins^29^,^35^,^36^, except for ScaC, which is more related to the type-I cohesins (dendrogram in Fig. 1). Nevertheless, sequence variations exist among the type-III cohesins. Therefore, the selected modules were chosen from different branches of the dendrogram. Putative cohesins, deemed too divergent from classic cohesins (namely ScaK, ScaL, ScaM1, ScaM2, ScaN and ScaP), were not selected for biochemical analysis.

### Identification of novel cohesin-dockerin interactions in *R. flavefaciens*

Unraveling the selective pattern of cohesin-dockerin binding within the *R. flavefaciens* cellulosome was achieved by employing three different approaches to detect protein-protein interactions. The three strategies are complementary and comprise cellulose-coated microarray, affinity-based ELISA assay, and *in-vivo* screening of co-expressed cohesin and dockerin modules with subsequent *in-vitro* validation by non-denaturing PAGE.

#### Microarray

Recombinant xylanase-fused dockerins (XynDocs) were interacted with CBM-fused cohesins (CBM-Coh). The latter allowed selective attachment to cellulose-coated slides^37^. The methodology was streamlined by applying crude cell extracts containing both CBM-Coh and XynDoc^38^, thereby facilitating analysis of large numbers of candidate modules.

**Figure 2:**
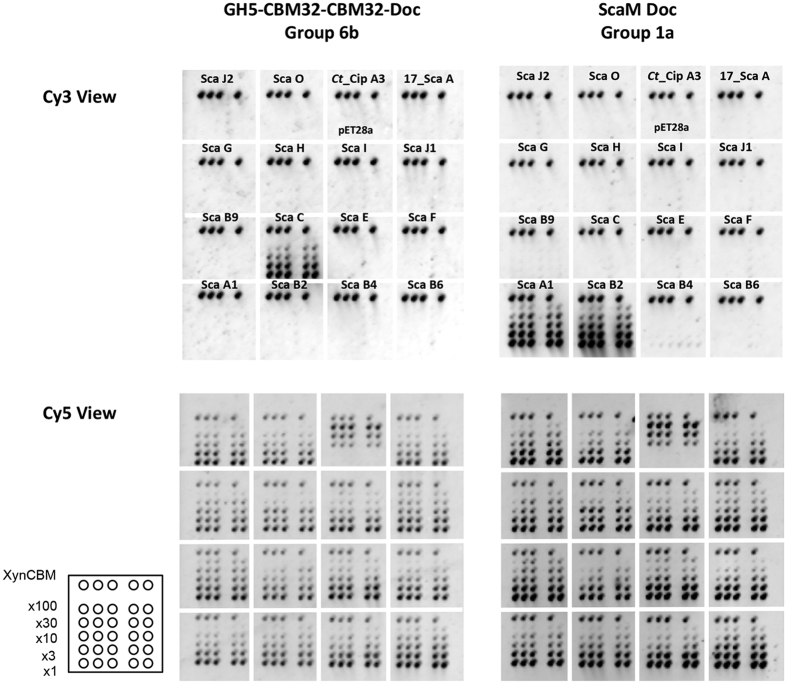
Representative cellulose-coated protein microarray screening, using crude cell extracts of both dockerin- and cohesin-fused proteins. XynDoc extracts derived from ScaM and a GH5 enzyme are shown as examples as probes against crude extracts of different CBM-cohesins, applied onto a cellulose-coated glass slide. Upper panel: Cy3-derivatized anti-Xyn antibody labeling revealed strong interaction of the group-6b GH5-borne dockerin and the ScaC cohesin (left), whereas the group-1a ScaM dockerin (right) interacted with ScaA cohesin 1 (A1) and ScaB cohesin 2 (B2). *C. thermocellum* CipA cohesin 3 (*Ct*_Cip A3) and the crude bacterial extract (transformed *E. coli* BL21 with an empty plasmid (pET28a) were used as negative controls. ScaA cohesin 3 of *R. flavefaciens* strain 17 (17_ScaA) was used to examine whether cross-strain interaction occurs. Lower panel: Cy5-derivatized anti-CBM antibody labeling observed for all of the printed protein spots on the microarray. The intensity of each spot is in linear correlation with the amount of CBM-Coh present. The array is divided into subarrays, each containing a different CBM-Coh sample. The top row of each subarray includes a XynCBM positive control, below which are serial dilutions by a factor of 3 of the crude cell extracts. Each CBM-Coh was printed in quintuplicate for each dilution. The scheme of all printed microarray samples is shown at the bottom left.

**Figure 3:**
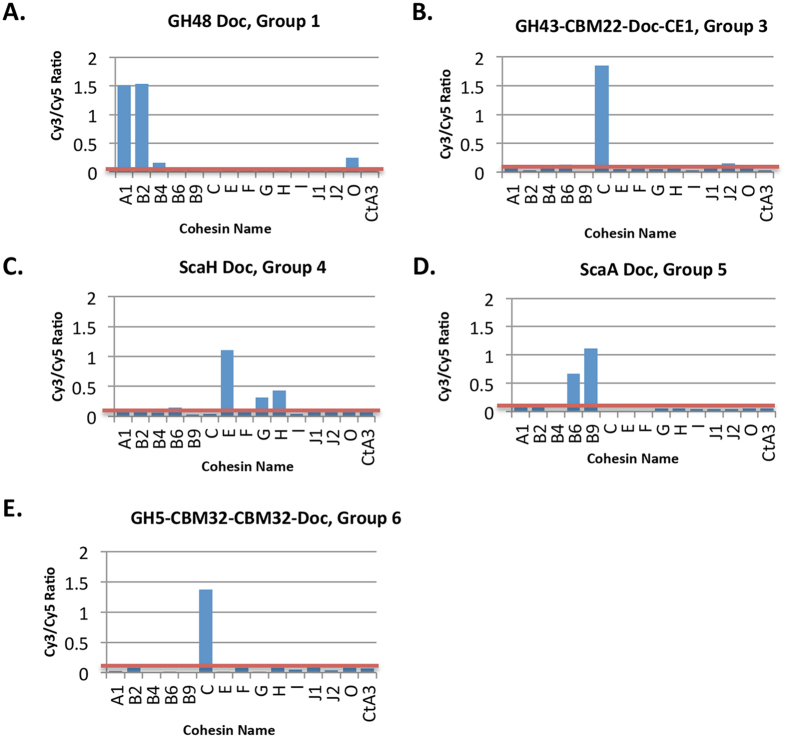
Quantification of representative interacting cohesin-dockerin pairs from *R. flavefaciens* strain FD-1 on cellulose-coated microarrays. Each bar graph represents interactions of a designated dockerin probe vs. 14 different cohesins (abscissa: ScaA1, ScaB2, ScaB4, etc.) and *C. thermocellum* CipA-CohA3 (*Ct*A3) as a control. (**A**) Group-1 dockerins, represented by ZP_06145360 (GH48 Doc). (**B**) Group-3 dockerins, represented by ZP_06141916 (GH43-CBM22-Doc-CE1). (**C**) Group-4 dockerins, represented by ZP_06142361 (ScaH-Doc). (**D**) The lone group-5 dockerin, ScaA-Doc (CAK18895). (**E**) Group-6 dockerins, represented by ZP_06143078 (GH5-CBM32-CBM32-Doc). See Table 1 for complete summary of the cohesin-dockerin interactions investigated in this work.

In Fig. 2, the data are presented for a series of representative CBM-Cohs applied to a cellulose-coated slide, subsequently interacted with a XynDoc probe (14 interactions tested per slide). The microarray technology was used to examine 14 *R. flavefaciens* cohesins (Fig. 1) and 32 dockerins (Table 1), yielding 448 possible interactions. Figure 3 shows representative interactions for different dockerin-containing scaffoldins and enzymes (in many cases, multi-functional). The data are shown as bar graphs taking into account non-specific background binding^39^. All reported binding levels were significantly above background. Note cohesin recognition trends delineate the different dockerin groups. Internal dockerins and N-terminal dockerins were as active as C-terminal dockerins. Curiously, most dockerins originating from LRR-containing parent proteins of the different groups did not interact with tested cohesins.

#### ELISA

The interaction of various *R. flavefaciens* recombinant XynDocs (Table 1) with CBM-Cohs was also tested using an ELISA approach. The binding of group-4 dockerins (i.e., ScaF, ScaH and ScaI dockerins, as well as peptidase-Doc) to ScaE, indicates that these components attach to the bacterial cell surface (Supplementary Figure S2). Several of these interactions displayed only weak binding using cellulose microarrays, yet IC_50_ indicate high-affinity binding (in the nano-molar range) of CttA XDoc, ScaH and ScaF, and an order-of-magnitude less for ScaI and peptidase-Doc. Based on these results, we concluded that such apparent low-affinity interactions, as revealed by the cellulose microarrays, should be regarded as possible positive hits, requiring further confirmation by complementary approaches.

#### *In-vivo* co-expression

**Figure 4:**
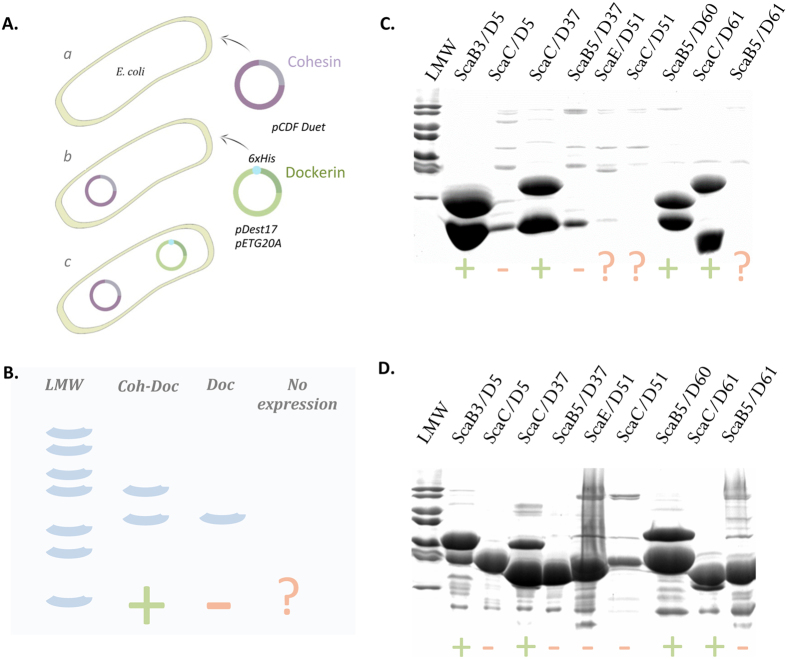
Identification of cohesin-dockerin complexes following recombinant *in-vivo* co-expression. (**A**) Schematic depiction of the recombinant *in-vivo* co-expression strategy. Cohesin-encoding genes were inserted into the pCDFDuet plasmid that was used to transform *E. coli* BL21(DE3) competent cells. Cells were made competent again and re-transformed with 45 Dockerins previously inserted into pDest17 (His-tag) and pETG20A (TrxA-His-tag). A total of 720 different clones (8 cohesins × 45 dockerins × 2 vectors) were obtained and used for co-expression. (**B**) Schematic illustration of the expected results. After purification by IMAC, *in-vivo* complex formation was evaluated by loading the purified samples onto SDS-PAGE gels. Since only the dockerins possessed a His tag, identification of complex formation was determined by the appearance of two bands in the gel, corresponding to the His-tagged dockerin and the bound cohesin. A single band corresponded to the isolated dockerin alone. The absence of bands indicated that the dockerin was either insoluble or did not express. (**C**). Representative experiment showing SDS-PAGE of selected samples: Two bands indicating *in-vivo* complex formation are clearly evident in the cases of ScaB3/D5 (group 1), ScaC/D37 (group 3), ScaB5/D60 (ScaADoc) and ScaC/D61 (group 6). Dockerin stability is greatly improved when bound to the cohesin as indicated by the difference in band intensity between bound and unbound dockerins. (**D**) Duplication of the experiment with TrxA-fused dockerins was carried out to eliminate false negatives due to low dockerin expression or insolubility. See Table 1 for complete summary of cohesin-dockerin interactions.

Dockerins are small unstable protein modules prone, to degradation when expressed in *E. coli*. However, recombinant dockerins are stabilized when bound to their counterpart cohesin. Thus, we devised a third complementary approach to identify novel cohesin-dockerin interactions within the *R. flavefaciens* cellulosome. Genes encoding different cohesin/dockerin partners were isolated and cloned into two compatible vectors for co-expression in *E. coli*. Recombinant dockerins contained an engineered N-terminal His tag. Immobilized metal-ion affinity chromatography (IMAC) was used to purify the recombinant dockerins together with the cohesins, upon binding between the two modules. Thus, protein complex formation was analyzed through SDS-PAGE by detecting the presence of a recombinant cohesin (Fig. 4A,B and Supplementary Figure S3). For these experiments 10 cohesins (Fig. 1) and 45 dockerins (Table 1) were selected. Initially, the capacity of recombinant *E. coli* strains to produce all 10 cohesins was evaluated. Two cohesins, from ScaG and ScaI, were insoluble when expressed under various conditions. Therefore, the *in vivo* expression studies were performed with the eight cohesins that expressed at detectable levels. Recombinant *E. coli* strains expressing the soluble cohesins were rendered competent and retransformed with 45 plasmids encoding dockerins. Since dockerins were expressed with either a single His-tag (in pDest17) or a thioredoxin fusion partner for increased solubility (pET20G), in total 720 interactions were tested (8 cohesins × 45 dockerins × 2 vectors). Analysis of the 720 recombinant strains, transformed with the cohesin- and dockerin-containing plasmids (exemplified in Fig. 4C,D) revealed that the capacity of *E. coli* to produce dockerins was severely impaired in the absence of a fusion protein (Fig. 4C). However, dockerin yield was significantly higher when a co-purified cohesin band was observed, confirming that binding to cohesin stabilizes dockerin structure leading to significant levels of protein production (Fig. 4D). Both co-expression experiments, using unfused and fused dockerins, generally revealed identical cohesin-dockerin specificity patterns. However, in some cases the size of the dockerin-fused protein was similar to that of the cohesin, making binding difficult to detect. Thus, the interaction of cohesin and dockerin pairs was validated by independent production of the two proteins in *E. coli*, using the TrxA-His fused dockerin derivative and His-tag fused cohesins. Following purification by IMAC, cohesin and dockerin modules were incubated to promote complexation, which allowed clarification of the cohesin-dockerin interactions.

### Novel cohesin-dockerin specificities reveal the overall architecture of the *R. flavefaciens* cellulosome

Data concerning the novel cohesin-dockerin specificities observed in *R. flavefaciens* cellulosomes, as evaluated by the three different platforms described above, are summarized in Table 1. In general, 5 major patterns of selectivity between cohesins and dockerins were observed, as follows:

- A broad range of group-1 dockerins recognized ScaA cohesins 1–2 and ScaB cohesins 2–4. Many of the dockerins in this group are components of enzymes, bearing catalytic motifs crucial for carbohydrate-degradation such as GHs in families 5, 9, 10, 11, 26, 43 and 48, which include the major cellulases and some hemicellulases; CEs from families 1, 3, 4 and 12) and CBMs. Some dockerins originate from established and putative cohesin-containing proteins, including ScaC, ScaE-like scaffoldin (ZP_06142991), ScaJ, ScaO, ScaM (Table 1).
- Both group-2 dockerins recognized the cohesins of ScaE and ScaH, as revealed by *in-vivo* co-expression and isothermal titration calorimetry (ITC) (see below).
- Dockerins of groups 3 and 6, exclusively recognized the same binding partner, the ScaC adaptor cohesin. Prior to the present work, only the dockerin of the enzyme CE3B (Table 1, Doc 31) was demonstrated to bind the ScaC cohesin^28^. This dockerin was included as a member of the group-3 dockerins^32^,^33^,^40^. Our study broads the range of possible interactions between the ScaC cohesin and dockerins belonging to groups 3 and 6. In this regard, the fact that the ScaC cohesin and dockerins of groups 3 and 6 share high sequence similarity with type I, and not type III, modules is of note^33^ (Fig. 1). This type of dockerin is almost exclusively a component of hemicellulases (GH families 5, 10, 11, 16, 24, 26, 43, 53 and 97), associated CEs, and some PLs.
- Similar to the group-2 dockerins, group-4 dockerins (notably those of CttA, ScaB, ScaF, ScaH, ScaI and peptidase-Doc) recognized the ScaE cohesin. Moreover, very weak binding of the CttA-XDoc and ScaH-Doc to cohesin H and the standalone cohesin G was observed in cellulose microarrays. The binding of group-4 dockerins to cohesins G and H was further supported by ELISA data, which provided evidence for ScaB-XDoc and ScaF-Doc as binding partners for these cohesins. Using the *in-vivo* screening approach, ScaH-Doc and another dockerin of a parent protein (ZP_06143271) of unknown function (UNK) were found to recognize cohesin H in addition to cohesin E. Interestingly, ScaH-Doc recognized its own cohesin. The ScaB and CttA dockerins were expressed with their adjacent upstream X-modules to ensure their functionality, as discussed previously^21^,^23^,^30^,^36^. As mentioned above, group-4 dockerins have a symmetrical sequence, as reflected by their two Ca^+2^-binding repeats, an apparent peculiarity for type III dockerin modules^33^. Further analysis of a possible dual-binding mode of group-4 dockerins by alanine scanning assay coupled with ELISA is detailed below.
- The unique ScaA dockerin is the only member of group 5. It was found to bind cohesins 5 through 9 on the ScaB scaffoldin, as formerly reported^41^,^42^,^43^.

### Probing the specificities of groups-2 and -4 dockerins and groups-3 and -6 dockerins by ITC

**Figure 5:**
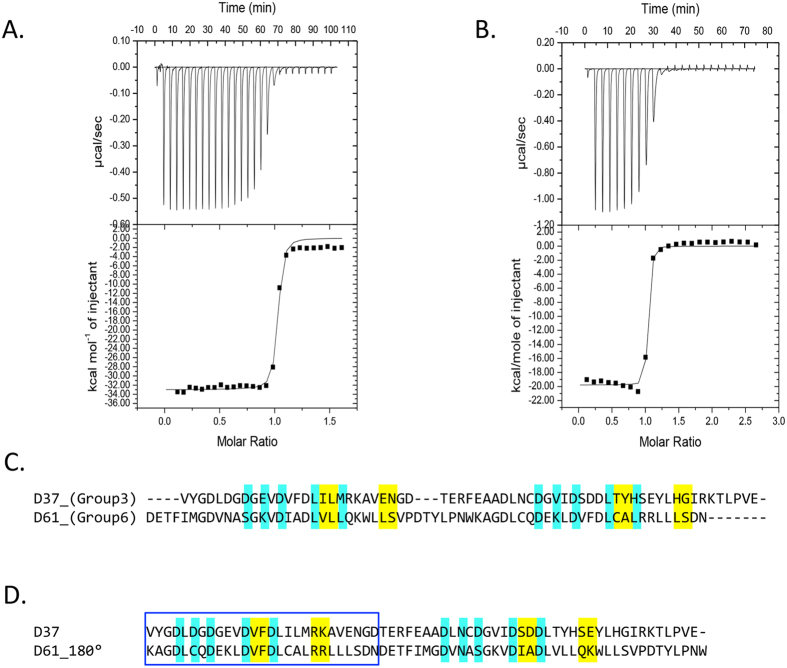
Binding of group-3 and group-6 dockerins to ScaC cohesin evaluated by ITC. The dockerins are numbered according to Table 1. Representative titrations are displayed in panel (A), ScaC Coh and dockerin 37 (D37), and (**B**), ScaC Coh and dockerin 61 (D61). The upper part of each panel shows the raw heats of binding, whereas the lower parts comprise the integrated heats after correction for heat dilution. The curve represents the best fit to a single-site binding model. (**D**) Alignment of dockerin D37 (group 3) with D61 (group 6) and of dockerin D37 with D61_180° (a mutated version of D61 in which the C-terminal half was switched with the N-terminal half). Note the similarity in the cohesin-recognition residues in the aligned first repeat (blue box, yellow highlight). Residues involved in Ca^+2^-binding are colored in cyan while putative residues involved in cohesin recognition are highlighted in yellow.

The data presented above suggest that dockerins of groups 3 and 6 bind exclusively to the ScaC cohesin. The interaction between representative members of groups-3 and -6 dockerins and ScaC cohesin was evaluated by ITC at 35 °C, the temperature of the *R. flavefaciens* microbial niche. The data (Fig. 5, Supplementary Table S2) reveal macromolecular association of high affinity (K_a_ 10^8^ M^−1^; stoichiometry of approximately 1:1). The sequences of these two dockerin groups indicate an asymmetric distribution of predicted recognition residues, suggesting a single-binding mode. When the two dockerins are aligned after swapping the C- and N-terminal halves of the group-6 dockerin, the identity at the putative cohesin-interacting region increases (Fig. 5D). A similar twofold alternative specificity mechanism was recently observed for cohesin-dockerin recognition in another ruminococcal species^44^.

Group-2 dockerins resemble truncated versions of group-4 modules^33^. ITC using representative members of groups-2 and -4 dockerins was performed to quantify the affinity of both interactions. Data, presented in Supplementary Figure S4 and Table S2, suggest a lower affinity constant (*K*_a_ of 10^6^–10^7^ M^−1^) compared with groups-3 and -6 dockerins. Alignments of groups-2 and -4 dockerins suggest that group-2 dockerins are highly homologous to the C-terminus of group-4 proteins (Fig. 4D and Supplementary Figure S5). ITC experiments also confirmed the affinity of group-2 dockerins to the ScaH cohesin (data not shown), although the interaction was too tight to accurately determine the *K*_a_ using this method. As described for other cohesin-dockerin pairs the interactions described here between *R. flavefaciens* cohesin-dockerin pairs are both enthalpically and entropically favorable^45^,^46^.

### Dual-binding mode in group-4 type III dockerins

**Figure 6:**
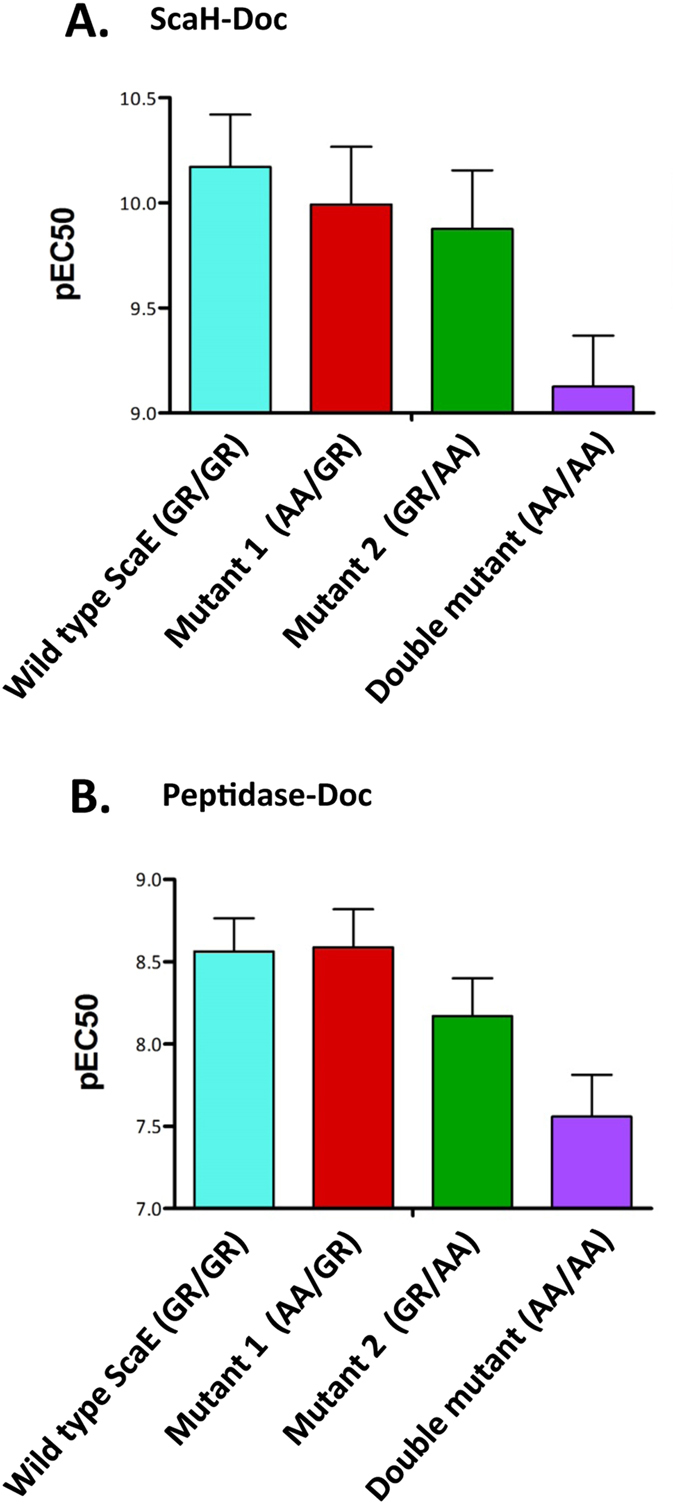
Dual-binding mode in the symmetrical group-4 dockerins. **(A**) ScaH Doc (ZP_06142361) and (**B**) peptidase-Doc (ZP_06142181). Alanine mutations were inserted at the major putative cohesin-recognition residues: positions G11/R12 and/or G50/R51, representing mutations in the first or second repeated segment of the dockerins, or the double mutant. Binding ability of the wild-type and mutants to the ScaE cohesin was examined by ELISA, and pEC50 values were determined as described previously^60^.

Data presented here suggest that group-4 dockerins associate to the bacterial cell envelope via recognition of the anchoring ScaE cohesin, without an upstream X-module and internal insertions^21^,^23^,^30^. Furthermore, these *R. flavefaciens* dockerins are generally distinctive within the realm of the type-III modules for their unique symmetrical nature. Alignment of these dockerins together with the XDocs of ScaB and CttA (Supplementary Figure S5) revealed that several of them, notably peptidase-Doc (ZP_06142181) and ScaH-Doc (ZP_06142361), exhibit similar Gly-Arg residues at postulated cohesin-recognition sites^23^,^47^. Interestingly, the dockerins of ScaB and CttA also possess duplicated Gly-Arg residues in both of their purported recognition sites, but the overall symmetry is disrupted by the characteristic extended insertions. Dockerins that exhibit symmetrical sequences have been shown in other bacterial species to possess two identical binding sites (i.e., dual-binding mode), thought to promote conformational flexibility to facilitate integration of enzymes into the cellulosomal complex and/or to overcome steric interactions which may interfere with the action of cellulosomal enzymes with the substrate^45^,^46^. To investigate such a role in *R. flavefaciens* strain FD-1, mutants of the above-designated symmetrical group-4 dockerins, containing Ala-Ala substitutions for the Gly-Arg dyad in one or both of the putative repeated recognition sites. From the extrapolated pEC50 values (Fig. 6), binding to the counterpart cohesin of ScaE was only impaired in the double mutant. Binding, however, was not completely eliminated due to apparent involvement of additional interacting residues. These results clearly indicate a dual-binding mode for the symmetrical group-4 dockerins.

## Discussion

**Figure 7:**
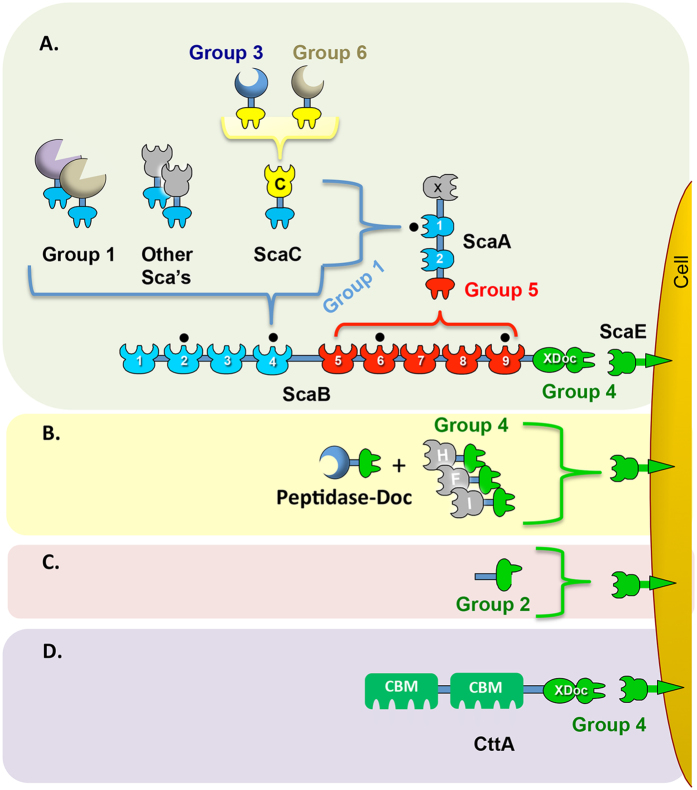
Current model of cellulosome assembly in *R. flavefaciens* strain FD-1. The scheme is color-coded to highlight the four subgroups of cohesin-dockerin specificities: Dockerins and cognate cohesin counterparts of the different groups are marked in light blue (Group-1 dockerins), yellow (Groups 3 and 6), green (Groups 2 and 4) and red (Group 5), respectively. The interacting partner(s) of cohesin modules marked gray, are yet to be discovered (and consequently yet to be confirmed as *bona fide* cohesins). (**A**) Cellulosomal proteins. (**B**) Cell wall-attached proteins. (**C**) Short (half) dockerins of group 2. (**D**) CttA subunit, purportedly mediating substrate attachment^23^.

The complexity of the *R. flavefaciens* FD-1 cellulosome system is reflected by its numerous secreted fiber-degrading dockerin-containing enzyme and non-enzymatic subunits and encoded scaffoldins, which can potentially generate innumerable configurations of cellulosome assemblies^29^,^33^,^34^. Using three experimental approaches to screen for cohesin-dockerin interactions, we accumulated evidence for several novel interactions between type III cohesins and their cognate dockerins belonging to heterogeneous groups. The results present recognition preference between the different cohesins and dockerins groups in this ruminal bacterium. They provide a snapshot of the molecular organization of the intricate *R. flavefaciens* cellulosome system, thus enabling routes of elaborate assembly of these multienzyme complexes, a model of which is proposed in Fig. 7.

The data correlate well with previous bioinformatic observations that *R. flavefaciens* dockerins exhibit exclusive sequence features allowing their classification into six distinct groups^33^. The second-order classification of the dockerin groups into eleven subgroups was found to be functionally redundant, since cohesin recognition among the various subgroups did not segregate with this subgroup classification. The subgrouping of these dockerin sequences may infer structural variations that reflect the stability of interaction with the cohesin or secondary interactions with the parent protein.

Borne *et al*.^48^ have recently demonstrated that, despite the general lack of interspecies cohesin-dockerin specificity, cellulosomes are not necessarily assembled in solution at random. The same study argued that enzyme binding to a cohesin will directly influence subsequent incorporation of other enzymes by mechanisms other than steric hindrance. These results support previous coarse-grain molecular modeling studies by Bomble *et al*.^49^. Moreover, preferential integration may also be related to inter-cohesin linker length^50^.

Group-1 dockerins comprise the majority of the encoded dockerins in the *R. flavefaciens* genome (96 ORFs) and mainly include multi-functional catalytic modules, such as numerous GHs, CEs, PLs and CBMs^29^,^34^. The data presented here support previous claims^28^ that Group-1 dockerins, whose sequence profile is exclusive to *R. flavefaciens*, preferentially bind cohesins ScaA1–2 and ScaB1–4.

Dockerins of groups 3 and 6 (mainly originating from hemicellulases) preferentially bound to the ScaC adaptor cohesin (Table 1). The common recognition profile suggests that enzymes associated with these dockerins might functionally interact. Interestingly, the putative recognition residues of these two dockerin groups are largely reversed, reminiscent of a similar phenomenon recently described for groups-3 and -4 dockerins of the human isolate *Ruminococcus champanellensis*^29^,^34^,^44^. Significantly, the ScaC cohesin is similar to type I cohesins of other cellulosome-producing bacteria, as opposed to the majority of type III cohesins in this bacterium.

Intriguingly, growth of *R. flavefaciens* strain 17 on xylan was shown to upregulate dockerin-containing enzymes that interact with the ScaC cohesin versus cultures grown on microcrystalline cellulose^32^. Moreover, the same study showed that components from cultures cultivated on xylan are enriched with very high-molecular-weight dockerin-bearing components that interact strongly with the ScaC Coh (and also with that of ScaA). In this context, high-molecular-weight multifunctional xylanases and carbohydrate esterases are produced by the various strains of *R. flavefaciens*^29^,^33^. The combined evidence suggests that ScaC may be involved in a regulatory mechanism that governs preferential expression of enzymes that act on hemicellulose.

A serpin-associated group-6 dockerin was also observed^33^. The serpin in this context may play a role in protecting the enormous cellulosome assembly from inadvertent extra-cellular proteolytic cleavage^51^,^52^. Such serpins also exist in other cellulosomal systems, such as those of *C. thermocellum* and *R. albus*^53^,^54^. Other putative roles could be regulatory in nature, since serpins are involved in cascade control processes or spatial confinement of developing signals^55^.

Previously, the ScaE cohesin had only been reported to interact with three proteins that share an X module-dockerin dyad: ScaB, CttA and a putative cysteine peptidase^23^,^47^. The well-characterized interactions of ScaB and CttA link the entire cellulosome machinery to the bacterial envelope and mediating substrate recognition and cell adhesion^21^,^23^,^28^. Single-molecule force spectroscopy revealed one of the strongest bimolecular protein-protein interactions yet reported for this type of interaction^56^. The dockerins possess three unique insertion regions that are absent in other dockerins. Recently, the crystal structure of the CttA-XDoc complex with ScaE was solved^30^,^31^, indicating that the insertions serve to reinforce the stalk like structure of the X module. Another form of X module was found to be involved in *C. thermocellum* type II interactions^57^. These modules are believed to contribute to the solubility, conformational state, structural and thermal stability and spatial flexibility of the cohesin-dockerin pair.

The dual-binding mode is proposed to decrease steric constraints imposed when multiple enzymes are integrated into a single scaffoldin unit, resulting, in some cases, in a bias towards cellulosome integration. Some *C. thermocellum* enzymes harbour unique type I dockerins, which are directed to the cell surface and appear to interact via a single-binding mode, since their pivotal cohesin-recognition residues at positions 11 and 12 of one of the dockerin-binding interfaces were atypical^58^. It was suggested that cellulosomal enzymes with dual-binding-mode dockerins may transiently interact with the bacterial cell surface before they are assembled into the multi-enzyme complexes. This mechanism would ensure retention by the bacterium even if cohesins are saturated. In addition, single-binding-mode dockerins recruit appended enzymes specifically to the cell surface. It is possible that synergism between cell surface-bound enzymes and cellulosomal enzymes may contribute to efficient hydrolysis of structural carbohydrates. Curiously, dockerin members of group 4 display internal symmetry of the two calcium-binding repeats, a phenomenon usually common to the majority of type I dockerins, but not prevalent in type III dockerins.

To summarize, this study has verified four major cohesin-dockerin recognition specificities in the cellulosome assembly of *R. flavefaciens* strain FD-1. Our findings provide an answer to the fundamental question whether bioinformatic classification of the 223 dockerin modules into groups with distinct sequence characteristics reflects binding specificity^33^. The data provided herein revealed the most complex and diverse cellulosome described to date. Not only does *R. flavefaciens* form the largest enzymatic consortium thus far identified, it also comprises the largest number of different cohesin-dockerin interactions observed in a single bacterium. This study demonstrates how a set of complimentary medium to high-throughput techniques can be applied to address functionally relevant questions concerning the activity of highly efficient nano-machines. We provide the basis for future exploration of novel cohesin-dockerin interactions in the field of nano-biotechnology, whereby recombinant chimeric scaffoldin constructs, harboring cohesins of different selective specificities, allow precise incorporation of matching dockerins attached to selected enzyme hybrids, thus promoting synergistic action of all biological processes that benefit from enzyme/protein proximity.

## Methods

### Protein microarrays

#### Cloning of cohesin and dockerin genes into fusion-protein expression cassettes

PCR primers were designed to amplify different dockerin- and cohesin-containing genes from the gDNA of *R. flavefaciens* strain FD-1^34^. A full list of primers is available in Supplementary Table S3. Constructs were prepared by standard molecular techniques. Briefly, dockerin inserts were cloned into the pET9d plasmid, supplemented with an N-terminal xylanase T-6 module, derived from *Geobacillus stearothermophilus*, and His-tag^59^. Cohesins were cloned into the pET28a plasmid, supplemented with an N-terminal family-3a carbohydrate-binding module (CBM3a) from CipA of *C. thermocellum*^60^. PCR reactions were conducted with Phusion DNA polymerase and DNA restriction reactions with Fermentas Fast Digest enzymes (ThermoFisher Scientific, Waltham, MA, USA). Preparation of xylanase-fused X-dockerins (XynDocs) of ScaB and CttA, and ScaE CBM-fused cohesin (CBM-Coh) were described earlier^59^,^60^. Plasmid DNA was extracted using a QIAprep Spin Miniprep Kit (Qiagen GmbH, Hilden, Germany). DNA integrity was confirmed by sequencing.

#### Recombinant expression of XynDoc and CBM-Coh fusion proteins

*E. coli* strain BL-21(λDE3) pLysS cells were used to over-express XynDoc and CBM-Coh fusion proteins as described^60^,^61^. XynDocs were incubated at 16 °C for 16 h; CBM-Cohs at 37 °C for 3 h, post induction.

#### Normalization of protein levels by SDS-PAGE

Whole-cell extracts of over-expressed CBM-Coh and XynDoc fusion proteins were examined on SDS-PAGE gels (12%), using ScaB2 CBM-Coh and ScaC XynDoc as standards, respectively.

#### Cy3- and Cy5-conjugated primary antibodies

Rabbit α-Xyn T6 and α-CBM antibodies were produced as described earlier^62^ and labeled with Cy3 and Cy5 mono-reactive dyes, respectively (GE Healthcare Bio-Sciences AB Uppsala, Sweden). Conjugates were dialyzed against Tris-buffered saline (137 mM NaCl, 2.7 mM KCl, 25 mM Tris pH 7.4; TBS).

#### Evaluation of Coh-Doc interactions upon cellulose-coated microarrays

The procedure was followed as documented^38^ with the following modification: Cohesin crude extracts were diluted 3-fold in TBS and printed in quintuplicate on cellulose slides. Nonspecific binding events were assessed using an unrelated cohesin (CipA-Coh3 of *C. thermocellum*), and crude extracts of transformed *E. coli* cells harboring an empty pET28a-CBM3a vector as negative controls. Fluorescent signal intensities were measured using ImageJ (http://imagej.nih.gov/ij). Following assignment of Cy3/Cy5 ratios, interactions were normalized according to a control XynCBM Cy3/Cy5 ratio of 1.

### Enzyme-linked immunosorbent assay (ELISA)

ELISA was conducted using XynDoc/CBM-Coh fusion protein pairs to evaluate cohesin-dockerin interactions as described^60^. DNA isolation/cloning and protein expression/purification were as above.

### *In-vivo* screening of cohesin-dockerin interactions

#### Cloning

Genes encoding 45 representative *R. flavefaciens* dockerins (Table 1) were cloned using the Gateway recombination cloning technology (ThermoFisher Scientific). Sequences were amplified by PCR using *R. flavefaciens* FD-1 genomic DNA as template and primers with engineered ends that allow site-specific recombination without need for restriction enzymes (Supplementary Table S4). Amplified genes were inserted into pDONR201 entry vector and subsequently into two distinct protein expression destination vectors, pDest17 and pETG-20A, according to the manufacturer’s protocol. In both expression vectors the genes are under T7 promoter control. pDest17 allowed fusion of N-terminal His-tags onto dockerins, while pETG-20A allowed fusion of N-terminal thioredoxin A with internal His-tag for increased stability/solubility. Genes encoding ten diverse cohesins were cloned into Novagen pCDFDuet vector (Merck Millipore, Darmstadt, Germany) using traditional restriction-enzyme methods. Cohesin sequences were isolated via PCR using genomic DNA as template. Primers incorporated 5′-NcoI and NheI restriction sites and 3′-XhoI (Supplementary Table S5). Digesting PCR products with NcoI and XhoI allowed cloning in the pCDFDuet vector, whereby engineered recombinant proteins contained no His-tag. Digesting with NheI and XhoI allowed cloning in pET21a to produce cohesins with C-terminal His-tags. Genes were sequenced in both directions to confirm that no mutations had occurred during amplification.

#### Detecting novel cohesin-dockerin complexes through *in vivo* co-expression

Cohesins and dockerins were co-expressed *in vivo* and co-purified by IMAC using the His-tagged dockerin. Initially, *E. coli* BL21 (DE3) cells were transformed with pCDFDuet poly-histidine tag lacking cohesins. Cohesin-harboring *E. coli* strains were made competent following conventional protocols. Each *E. coli* pCDFDuet-cohesin was retransformed with pDest17 and pETG20-A derivatives encoding the 45 dockerins. The two plasmids, pCDFDuet and either pDest17 or pETG20-A, have compatible origins of replication and independent antibiotic selection, leading to a total of 720 different recombinant *E. coli* strains expressing 720 cohesin-dockerin combinations. Cells transformed with the two plasmids were used to inoculate 5 ml of LB media with Ampicillin and Streptomycin in deep-well plates and grown to OD_600_ of approximately 0.4–0.6. Expression was induced by 1 mM of IPTG, and cells were grown an additional 16 h at 19 °C before harvesting. Cell pellets were then re-suspended in 1 ml lysozyme-containing buffer (8.4 mM 4-(2-hydroxyethyl)-1-piperazineethanesulfonic acid (HEPES), pH 7.5, 10 mM imidazole, 167 mM NaCl, 0.83 mM CaCl_2_ and 0.25 mg/mL lysozyme) and kept at −80 °C for 1 h. IMAC was performed in 96-well plates using a manifold vacuum system (Merck Millipore, Darmstadt, Germany). Purified samples were then subjected to 14% SDS-PAGE, and visualization of either two (cohesin + His tagged dockerin complex), one (His-tagged dockerin) or no bands (no expression) was annotated.

#### Probing cohesin-dockerin interactions by native PAGE

For non-denaturing PAGE assays, cohesins and dockerins were expressed and purified independently. pET21a plasmid derivatives encoding cohesins were used to transform BL21 (DE3) cells. The cells were used to inoculate 200-ml LB media with Ampicillin and grown to OD_600_ of approximately 0.4–0.6. Expression was induced by IPTG as above. Cells were harvested and kept at −20 °C for 1 h, lysed by ultra-sonication in 50 mM HEPES buffer, pH 7.5, containing 1-M NaCl, 5 mM CaCl_2_, 10 mM imidazole. Protein purification was performed through IMAC in 1-ml His GraviTrap gravity flow columns (GE Healthcare, Little Chalfont, Buckinghamshire, UK). Dockerins were expressed in deep-well plates and purified with the manifold vacuum system. Each cohesin was incubated with each dockerin (25 μM of each module in elution buffer) for 30 min. Samples of the isolated dockerins, cohesins and respective mixtures were examined by non-denaturing PAGE.

### Isothermal titration calorimetry (ITC)

Affinity and thermodynamics of representative cohesin-dockerin interactions was evaluated by ITC. Recombinant cohesins (pET21a vector; containing a His-tag) and dockerins (pETG20A vector; containing an N-terminal thioredoxin-His tag) were produced separately and purified by IMAC. Proteins were buffer exchanged by PD-10 Sephadex G-25M gel filtration (GE Healthcare) columns into 50-mM Na-HEPES buffer, pH 7.5, containing 2 mM CaCl_2_ and 0.5 mM tris(2-carboxyethyl)phosphine (TCEP). Briefly, thioredoxin-fused dockerins (20–30 μM) were stirred at 307 rpm in the reaction cell, injected with 10-μL aliquots of 80–180 μM cohesin solution at 220-s intervals. Titrations were performed at 308.16 K. Integrated heat effects, after correction for heats of dilution, were analyzed by nonlinear regression using a single-site model (Microcal ORIGIN version 7.0, Microcal Software, Northampton, MA). The fitted data yielded the association constant (*K*_*A*_) and enthalpy of binding (*ΔH*). Other thermodynamic parameters were calculated by the standard thermodynamic equation: *ΔRT*ln*K*_*A*_ = *ΔG* = *ΔH* − *TΔS.*

### Alanine-scanning assay

The two-fold symmetry observed in some group-4 dockerin sequences renders them similar to those of type I, previously shown to exhibit dual-binding mode^45^,^46^. Consequently, putative group-4-dockerin recognition residues of cysteine peptidase (ZP_06142181) and ScaH (ZP_06142361) were chosen for alanine-scanning, based on sequence similarity to ScaB and CttA XDocs in their presumed cohesin-recognition residues (Supplementary Figure S5). To substitute two amino acids simultaneously, overlap-extension PCR was conducted^63^. Thus, in two sequential PCR reactions exploiting two sets of primers (Supplementary Table S6), double Ala mutations were introduced into the first Ca^+2^-binding loop and the third helix of the dockerins instead of the original Gly-Arg residues (positions 10–11 and 48–49). The resultant three variants included a mutant carrying Ala-Ala in positions 11–12 of the first dockerin repeat (mutant 1), a mutant carrying Ala-Ala in positions 48–49 (mutant 2) of the second dockerin repeat and a variant harboring both sites of mutations (double mutant). Mutated XynDocs were expressed and purified by IMAC. Interaction between each XynDoc and CBM-CohE was evaluated by ELISA and further processed as described^60^.

## Additional Information

**How to cite this article**: Israeli-Ruimy, V. *et al*. Complexity of the *Ruminococcus flavefaciens* FD-1 cellulosome reflects an expansion of family-related protein-protein interactions. *Sci. Rep.* **7**, 42355; doi: 10.1038/srep42355 (2017).

**Publisher's note:** Springer Nature remains neutral with regard to jurisdictional claims in published maps and institutional affiliations.

## Supplementary Material

Supplementary Material

## Figures and Tables

**Table 1:** Summary of interacting *R. flavefaciens* FD-1 cohesin and dockerin modules depicted by the various strategies used in this work: Cellulose-coated microarrays, ELISA, and *in-vivo* screening followed by non-denaturing PAGE.

	Accession no.	Group No.	Cohesin Architecture of parental-enzyme	A1	B2	B3	B4	B5-B9	C	E	G	H
1	ZP_06141990	1a	UNK-**Doc**	+		+		−	−	−		−
2	ZP_06142678	1a	GH9-CBM3-**Doc**	+	+	+	+	−	−	−	−	−
3	ZP_06143384	1a	GH44-**Doc**	+		+		−	−	−	−	−
4	ZP_06143935	1a	LRR-**Doc**	+		+		−	−	−		−
5	ZP_06144449	1a	UNK-CE12-CBM13-**Doc**-CBM35-CE12	+		+		−	−	−		−
6	ZP_06145345	1a	UNK-**Doc**	+		+		−	−	−		−
7	ZP_06145412	1a	LRR-**Doc**	+		+		−	−	−		−
8	ZP_06145411	1a	GH5-**Doc**	+		+		−	−	−	−	−
9	ZP_06145755	1a	GH5-**Doc**	+		+		−	−	−	−	−
10	ZP_06144897	1a	UNK-**Doc**	+		+		−	−	−	−	-
11	ZP_06142769	1a	GH11-CBM22-GH10-**Doc**-CBM22-CE4	+	+		−	−	−	−	−	−
12	ZP_06142857	1a	GH11-CBM22-**Doc**-GH11-CE3	+	+		−	−	−	−	−	−
13	ZP_06142983	1a	UNK-CE12-CBM13-**Doc**-CBM35-CE12	+	+		+	−	−	−	−	−
14	ZP_06145360	1a	GH48-**Doc**	+	+		−	−	−	−	−	−
15	ZP_06144535	1a	Coh-**Doc** (ScaO)	+	+		−	−	−	−	−	−
16	ZP_06145505	1a	Coh-**Doc** (ScaM)	+	+		−	−	−	−	−	−
17	ZP_06141671	1b	CBM-GH9-**Doc**	+		+		−	−	−		−
18	ZP_06144353	1b	LRR-**Doc**	+		+		−	−	−		−
19	CAK18894	1b	Coh-**Doc** (ScaC)	*	*	*	*	−	−	−	−	−
20	ZP_06141810	1b	UNK-**Doc**	+		+		−	−	−	−	−
21	ZP_06142866	1b	GH9-UNK(CBM?)-UNK(CBM?)-**Doc**	+	+		+	−	−	−	−	−
22	ZP_06145705	1b	GH43-UNK-CBM13-CBM13-**Doc**	+	+		+	−	−	−	−	−
23	ZP_06142105	1c	UNK-LamGL(CBM?)-**Doc**	+	+		+	−	−	−	−	−
24	ZP_06142374	1d	UNK-**Doc**	+		+		−	−	−		−
25	ZP_06144548	1d	UNK-**Doc**-UNK	+		+		−	−	−		−
26	ZP_06145497	1d	Coh-Coh-**Doc** (ScaJ)	+	+	+	−	−	−	−	−	−
27	ZP_06144651	2	LRR-**Doc**	−				−		+		+
28	ZP_06143271	2	UNK-**Doc**-UNK	−				−		+		+
29	ZP_06143424	3	PL-CBM-**Doc**	−				−	+	−		−
30	ZP_06145446	3	CBM4-GH10-CBM9-**Doc**	−		−		−	+	−	−	−
31	ZP_06143878	3	CE-CBM-**Doc**-UNK (known as “CE3B”)	−		−		−	*	−	−	−
32	ZP_06141916	3	GH43-X19-CBM22-**Doc**-CE1	−	−	−	−	−	+	−	−	−
33	ZP_06143260	3	GH53-CE-**Doc**	−		−		−	+	−	−	−
34	ZP_06142964	3	UNK-**Doc**						+			
35	ZP_06144896	3	GH11-UNK-**Doc**	−	−		−	−	+	−	−	−
36	CAK18896	4a	Coh-Coh-Coh-Coh-Coh-Coh-Coh-Coh-Coh-**X-Doc** (ScaB)	−	−	−	−	−	−	*	−+	−+
37	CAK18897	4a	CBM-CBM-**X-Doc** (CttA)	−	−	−	−	−	−	*	+	+
38	ZP_06142651	4a	UNK-**Doc**	−		−		−	−	+		
39	ZP_06142361	4a	Coh-**Doc** (ScaH)	−	−	−	−	−	−	+	+	+
40	ZP_06144588	4a	Coh-**Doc** (ScaF)	−	−	−	−	−	−	+	−+	−+
41	ZP_06142181	4a	Peptidase-UNK-**Doc**	−	−		−	−	−	+	−	−
42	ZP_06143695	4a	UNK-**Doc**	−				−	−	+		+
43	ZP_06145744	4a	LRR-Coh-**Doc** (ScaI)	−	−		−	−	−	+	−	−
44	CAK18895	5	UNK-Coh-Coh-**Doc** (ScaA)	−	−	−	−	*	−	−	−	−
45	ZP_06142459	6a	“zincins”-**Doc**-UNK	−				−	+	−		−
46	ZP_06144432	6a	UNK-**Doc**	−				−	+	−		−
47	ZP_06145118	6a	GH18-**Doc**	−				−	+	−		−
48	ZP_06142855	6a	UNK-PL-**Doc**	−		−		−	+	−	−	−
49	ZP_06143179	6a	UNK-PL-**Doc**	−		−		−	+	−	−	−
50	ZP_06143476	6a	UNK(LbetaH-LamGL)-**Doc**	−	−		−	−	+	−	−	−
51	ZP_06142906	6b	**Doc**-Serpin	−	−		−	−	+	−	−	−
52	ZP_06144185	6b	UNK-LRR-Cysteine proteinase-**Doc**	−				−	+	−		
53	ZP_06143078	6b	GH5-CBM32-CBM32-**Doc**	−	−		−	−	+	−	−	−

Accession numbers, architecture of the dockerin-bearing parent proteins and group classification (see also Supplementary Figure S1) are designated.

The dockerin module is marked in boldface for each ORF.

Dockerins 1–16, 17–22, 23, 24–26, 27–28, 29–35, 36–43, 44, 45–50, 51–53 represent dockerin groups: 1a, 1b, 1c, 1d, 2, 3, 4a, 5, 6a and 6b, respectively. Twenty-four dockerins that were cloned and expressed but did not exhibit any interaction are available in Table S1. Glycoside hydrolase families 5, 9, 44 and 48 are putative cellulases and families 10, 11 and 43 are putative xylanases.

Key to symbols in the Table:

+ Novel interactions discovered in the present study.

* Previously reported interactions.

− Interactions examined but found to be negative.

Untested pairs by the designated methods.
