# Supplementary material for: Complexity of the Ruminococcus flavefaciens FD-1 cellulosome reflects an expansion of family-related protein-protein interactions
Source: Sci Rep. 2017 Feb 10;7:42355. doi: 10.1038/srep42355 (PMC5301203; doi:10.1038/srep42355)
Supplement: Supplementary Material [file srep42355-s1.pdf]

Complexity of the *Ruminococcus flavefaciens* FD-1 cellulosome reflects an expansion of family-related protein-protein interactions

Vered Israeli-Ruimy[1,*], Pedro Bule[2,*], Sadanari Jindou[3†], Bareket Dassa[1], Sarah Moraïs[1], Ilya Borovok[3], Yoav Barak[1,4], Michal Slutzki[1], Yuval Hamberg[1], Vânia Cardoso[2], Victor D. Alves[2], Shabir Najmudin[2], Bryan A. White[5,6], Harry J. Flint[7], Harry J. Gilbert[8], Raphael Lamed[3], Carlos M.G.A. Fontes[2] and Edward A. Bayer[1]**

Supplementary Material

Supplementary Tables

Table S1. List of non-interacting dockerin modules, tested by the various strategies in this work. The last 3 lanes correspond to the three methods used: Cellulose-coated microarray (CM), ELISA (E), and recombinant *in-vivo* co-expression (Iv). Dockerins tested using the designated method were marked (X). Italicized *X* indicates that the given dockerin was insoluble.

	Accession No.	Group	Architecture of parental enzyme	CM	E	Iv
1	ZP_06144474	1a	UNK-Doc	X		
2	ZP_06142991	1b	Coh-Doc (ScaE-like)	X		
3	ZP_06144783	1c	UNK-Doc-UNK	X		
4	ZP_06143931	1c	UNK-Doc			X
5	ZP_06143761	1d	UNK-Doc	X		
6	ZP_06141956	3	Doc-UNK			X
7	ZP_06143670	3	Coh-Doc (ScaL)	X		
8	ZP_06143567	4a	LRR-Doc-UNK	X		
9	ZP_06143379	4a	Doc-UNK-GH3			*X*
10	ZP_06142016	4a	UNK-Doc			X
11	ZP_06144357	4a	Doc-UNK			X
12	ZP_06145754	4a	Doc-UNK			X
13	ZP_06142815	4b	UNK-Doc		X	X
14	ZP_06142816	4b	LRR-Doc	X		
15	ZP_06143103	6a	GH43-Doc		X	
16	ZP_06142225	6b	UNK-PL-UNK-Doc			X
17	ZP_06143324	6b	Doc-UNK			*X*
18	ZP_06142338	Unclassified	UNK-CBM13-Doc-GH43-UNK-GH43-UNK			X
19	ZP_06142740	Unclassified	UKN-Doc-UNK			*X*
20	ZP_06142981	Unclassified	UKN-Doc-UNK-Doc-UNK			*X*
21	ZP_06144059	Unclassified	UKN-Doc			X
22	ZP_06145331	Unclassified	GH11-CBM22-GH10-Doc-GH11-CE4			*X*

Table S2 Thermodynamics of novel cohesin-dockerin interactions identified in *R. flavefaciens* cellulosome as evaluated by ITC. Thermodynamic parameters were determined at 308.16 K.

Interaction	K_a M^{-1}	ΔG^o *kcal mol^{-1}*	ΔH *kcal mol^{-1}*	$T\Delta S^o$ *kcal mol^{-1}*
ScaCCoh/D32 (Group 3)	2.69E8 ± 2.52E7	-11.85	-36.33 ± 0.055	-24,48
ScaCCoh/D53 (Group 6)	3.54E8 ± 1.37E7	-12.05	-19.79 ± 0.183	-7.73
ScaECoh/D30 (Group 2)	6.17E7 ± 3.13E6	-10.99	-23.00 ± 0.520	-12.01
ScaECoh/ScaHDoc (Group 4)	1.56E6 ± 1.61E5	-5,90	-64.11 ± 1.181	-58.21

Supplementary Table S3. Dockerin modules of *R. flavefaciens* strain FD-1 selected for the microarray study.

Accession No.	Group	Architecture of parent protein	Primers used
ZP_06142678	1a	SIGN-GH9-CBM3-Doc	5' gctac**ggtacc**t GAG CGT GTT ACT CTG TGG<br>3' cgccag***ggatcc***TTA TCA GTT ATA GCT CTC GGG
ZP_06142769	1a	SIGN-GH11-CBM22-GH10-Doc-CBM22-CE4	5' gctac**ggtacc**t GTA ACA CTC TGG GGC GAT GCT<br>3' cgccag***ggatcc*** TTA TGC GAT ATA TGT CTT ATT TGA TGC
ZP_06142857	1a	SIGN-GH11-CBM22-Doc-GH11-CE3	5' gctac**ggtacc**t ACA CTC TGG GGC GAT GCC<br>3' cgccag***ggatcc*** TTA CTG ATA ATT TGA TCT TGA GGC
ZP_06142983	1a	SIGN-UNK-CE12-CBM13-Doc-CBM35-CE12	5' gctac**ggtacc**t GAG GCT GTT CAG AAG TTC<br>3' cgccag***ggatcc***TTA TTC GGG CTC ATA GTA AAC
ZP_06144535	1a	SIGN-Coh- Doc (ScaO)	5' gctac**ggtacc**t TCT GTA ACT TCA ACA GTC AAA G<br>3' cgccag***ggatcc*** TTAACT CTC CAC AAA CTC CCA GT
ZP_06145360	1a	SIGN-GH48-Doc	5' gctac**ggtacc**t GTT CTC TGG GGC GAT GCT<br>3' cgccag***ggatcc***TTA TGA CTC AGG GAG CTT AGT
ZP_06145505	1a	SIGN-Coh-Doc (ScaM)	5' gctac**ggtacc**t TTA GAG ATA GTT CTT GAT GAA CC<br>3' cgccagggatccTTA ATC AAG CTT CAG CAG TTT TTT C
ZP_06142866	1b	SIGN-GH9-UNK(CBM?)-UNK(CBM?)-Doc	5' gctac**ggtacc**t GCT ACT ATC GTT GGT GAC<br>3' cgccag***ggatcc***TTA TTA CTT AGT TGT TGG GAG AG
ZP_06142991	1b	SIGN-Coh-Doc (ScaE-like)	5' gctac**ggtacc**t GTC GGC GAC TAC AAT GCA<br>3' cgccag***ggatcc*** TTA ATC TTC GGG GAG CGA AGG
ZP_06145705	1b	SIGN-GH43-UNK-CBM13-CBM13-Doc	5' gctac**ggtacc**t GGA CTT GCA GGC GAT ACC<br>3' cgccag***ggatcc***TTA TCA GCT TGT CAG CTT GTC
CAK18894	1b	SIGN-Coh-Doc (ScaC)	5' gctac**ggtacc**t CCC GAT CAG GCT ACT CTG<br>3' cgccag***ggatcc*** TTA TCA AAG TTC TGT GAT GAG AG
ZP_06142105	1c	SIGN-UNK-LamGL(CBM?)-Doc	5' gctac**ggtacc**t GCC GGT ATT CTC TGG GGC<br>3' cgccag***ggatcc*** TTA TTA TTT GCT ATA GGA TTC GGG
ZP_06145497	1d	SIGN-Coh-Coh-Doc (ScaJ)	5' gctac**ggtacc**t ACT GCT GCT GAG CCT GTA<br>3' cgccag***ggatcc*** TTA ATG TCA TTA TTC AAG CTT CAG
ZP_06141916	3	SIGN-GH43-X19-CBM22-Doc-CE1	5' gctac**ggtacc**t TCC GGT GAC GTT CAG TAT ATC<br>3' cgccag***ggatcc*** TTA GGC AGG CTG ACT TTC TCC
ZP_06144896	3	SIGN-GH11-UNK-Doc	5' gctac**ggtacc**t TAT GAG ATC ATG GGT GAC<br>3' cgccag***ggatcc*** TTA CTT TTG GGA AGC CTT GTC
ZP_06142181	4a	SIGN-Peptidase-UNK-Doc	5' gctac**ggtacc**t CTC ACA CTG CTT CTG AAA CGT<br>3' cgccag***ggatcc*** TTA CTA ATT TAT TAC AGA TGA TTT AGC
ZP_06142361	4a	SIGN-Coh-Doc (ScaH)	5' gctac**ggtacc**t AAA CCG CAG TAC CGC CTC<br>3' cgccag***ggatcc*** TTA TCA ACC TCT GAG AGG CTG
CAK18896	4a	SIGN-Coh-Coh-Coh-Coh-Coh-Coh-Coh-Coh-UNK-Coh-UNK-Doc (ScaB)	5' aatt**ggtacc**aACTACAGCAACAAATTCCGGTG<br>3' taat***ggatcc***TTAACCGAATCTGTTTGGAAC
CAK18897	4a	SIGN-CBM-CBM-Doc (CttA)	5' aatt**ggtacc**aAACACTGTTACATCAGCTG<br>3' ttaa***ggatcc***TTATTCTTCTTCAGCATCGCC
ZP_06144588	4a	SIGN-Coh-Doc (ScaF)	5' gctac**ggtacc**t GAT GAA ACT ACT GAG TAT AAG<br>3' cgccag***ggatcc*** TTA TGG AGA ATT ATG AGC CTG
ZP_06145744	4a	SIGN-LRR-Coh-Doc (ScaI)	5' gctac**ggtacc**t GCG GTT ATT ATC GGC GAT<br>3' cgccag***ggatcc*** TTA TCT GCT TGC GTT TAT AAA TTC
CAK18895	5	SIGN-UNK-Coh-Coh-Doc (ScaA)	5' gctac**ggtacc**t CCA AGC GGC AAC ACA CTC<br>3' cgccag***ggatcc***TTA TTA GCC CTT AGC AGG GAG
ZP_06143476	6a	SIGN-UNK(LbetaH-LamGL)-Doc	5' gctac**ggtacc**t GAA GCA GAC AGT TTC ATT ATG<br>3' cgccag***ggatcc***TTA TTA TTG TTT CAG AAG TTC ACG
ZP_06142906	6b	SIGN-Doc-SERPIN	5' gctac**ggtacc**t GCT CTC GAA CCG CCA AGG<br>3' cgccag***ggatcc***TTA AGG ATG AGC GCT TTC AAT GCC
ZP_06143078	6b	SIGN-GH5-CBM32-CBM32-Doc	5' gctac**ggtacc**t GGA CAG AAA TCA GCT GAG<br>3' cgccag***ggatcc***TTA TTA TTT GTT GAG TAT TTT TCT GAG

Supplementary Table S4. Dockerin modules of *R. flavefaciens* strain FD-1 selected for the *in vivo* study.

Accession No.	Group	Architecture of parent protein	Primers used
ZP_06141990	1a	UNK-Doc	5' ggggacaagtttgtacaaaaaagcaggcttc TCA GAA TAT TCC GCA CCT GTC<br>3' ggggaccactttgtacaagaaagctgggtc TTA TAA GCC GAG CAG TTT CAT CTG
ZP_06142678	1a	SIGN-GH9-UNK-CBM3_1-LNK-Doc	5' ggggacaagtttgtacaaaaaagcaggcttc GTT ACT CTG TGG GGA GAC GCT AAC<br>3' ggggaccactttgtacaagaaagctgggtc TCA GTT ATA GCT CTC GGG AAG CTC
ZP_06143384	1a	SIGN-UNK-GH44-UNK-LNK-Doc	5' ggggacaagtttgtacaaaaaagcaggcttc CCC GCA AAC GTA ACA TAC GGC<br>3' ggggaccactttgtacaagaaagctgggtc TTA TGC TTC GGG AAG CTT GTC
ZP_06143935	1a	SIGN-UNK-X159-X159-UNK-Doc	5' ggggacaagtttgtacaaaaaagcaggcttc CCG AAA CCG GAT CTT ACC GGT GAC<br>3' ggggaccactttgtacaagaaagctgggtc TTA TTT CTT CTC GGG TAA TTC GG
ZP_06144449	1a	SIGN-X70-CE12-CBM13-LNK-Doc-LNK-CBM35-CE12	5' ggggacaagtttgtacaaaaaagcaggcttc GAG GCT GTT CAG AAG TTC CCG GG<br>3' ggggaccactttgtacaagaaagctgggtc TCA AGC GGG CTC TAC CGG CTG TTT AG
ZP_06145345	1a	SIGN-UNK-Doc	5' ggggacaagtttgtacaaaaaagcaggcttc AAA GTT TCA GAA GTA AAG GGT GAC<br>3' ggggaccactttgtacaagaaagctgggtc TTA TAC GAG CTT GAG GAG GAT C
ZP_06145412	1a	SIGN-UNK-X159-X159-UNK-X159-X159-X159-X159-X159-X159-UNK-UNK-X159-X159-X159-UNK-X159-X159-X159-UNK-X159-X159-X159-UNK-Doc	5' ggggacaagtttgtacaaaaaagcaggcttc TAC GGC GAC GCT AAC CTT GAC<br>3' ggggaccactttgtacaagaaagctgggtc TTA TTC TTT ATC GGG AAG TGT GG
ZP_06141671	1a	SIGN-CBM4-X229-GH9-LNK-Doc	5' ggggacaagtttgtacaaaaaagcaggcttc AAT GTT ACT CTC TGG GGC GAC<br>3' ggggaccactttgtacaagaaagctgggtc TCA CTC TGG AAG ATT TCC GAT AAG
ZP_06142866	1b	SIGN-UNK.GH9-UNK-LNK-Doc	5' ggggacaagtttgtacaaaaaagcaggcttc ATC GTT GGT GAC GCT AAC TGC<br>3' ggggaccactttgtacaagaaagctgggtc TTA CTT AGT TGT TGG GAG AGT TG
ZP_06142991	1b	SIGN-Coh-Doc (ScaG)	5' ggggacaagtttgtacaaaaaagcaggcttc GTC GGC GAC TAC AAT GCA GGC<br>3' ggggaccactttgtacaagaaagctgggtc TTA ATC TTC GGG GAG CGA AGG
ZP_06144353	1b	SIGN-UNK-Doc	5' ggggacaagtttgtacaaaaaagcaggcttc GAT CAG GCT ACT CTG AGA GGC<br>3' ggggaccactttgtacaagaaagctgggtc TCA AAG TTC TGT GAT GTC
ZP_06144572	1b	SIGN-Coh-UNK-Doc (ScaC)	5' ggggacaagtttgtacaaaaaagcaggcttc GTT TCA GAA AAT GTA AAT GGC<br>3' ggggaccactttgtacaagaaagctgggtc TCA CTG CTC AAT ATC ATC TTT TAT ACC
ZP_06145705	1b	SIGN-UNK-GH43-UNK-CBM13-CBM13-LNK-Doc	5' ggggacaagtttgtacaaaaaagcaggcttc GAA GAA CAG GGA CTT GCA GG<br>3' ggggaccactttgtacaagaaagctgggtc TCA GCT TGT CAG CTT GTC AAC
ZP_06143931	1c	SIGN-UNK-Doc	5' ggggacaagtttgtacaaaaaagcaggcttc ATT ATA AAC GGC ATT GAA GGC<br>3' ggggaccactttgtacaagaaagctgggtc TCA GTC AAG CTT CAG CAG
ZP_06142374	1d	SIGN-UNK-Doc	5' ggggacaagtttgtacaaaaaagcaggcttc GCA TTG AAA ACT AAT AGT ATC<br>3' ggggaccactttgtacaagaaagctgggtc TTA TTC AAG CTT CAG CAG
ZP_06144548	1d	SIGN-UNK-Doc-UNK	5' ggggacaagtttgtacaaaaaagcaggcttc ACT GAC AGT GTA TTA TAC GGT GAC<br>3' ggggaccactttgtacaagaaagctgggtc TCA TAT ATC AGC AGC ATC ATT CAG
ZP_06145497	1d	SIGN-UNK-Doc (ScaJ)	5' ggggacaagtttgtacaaaaaagcaggcttc GCT GCT GAG CCT GTA AAT GGC<br>3' ggggaccactttgtacaagaaagctgggtc GTC TTA TTC AAG CTT CAG CAG
ZP_06143271	2	SIGN-UNK-LNK-Doc-LNK-UNK	5' ggggacaagtttgtacaaaaaagcaggcttc GGC GAT ATC AAC GGC GAT GGT ATC<br>3' ggggaccactttgtacaagaaagctgggtc TCA TGT TGT GGT ATC TTC AGC
ZP_06141956	3	SIGN-Doc-UNK	5' ggggacaagtttgtacaaaaaagcaggcttc GAT ATC CTC ACA CTT TTC GGC<br>3' ggggaccactttgtacaagaaagctgggtc TCA AAG GGT TCC GCC GAC GGG
ZP_06142964	3	X231-UNK-Doc	5' Ggggacaagtttgtacaaaaaagcaggcttc GGC GAT ATA AAC CTT GAC GGC<br>3' ggggaccactttgtacaagaaagctgggtc TTA TCC TAT AAG CAT TTT GCG
ZP_06143424	3	SIGN-X141-CBM6-Doc1	5' ggggacaagtttgtacaaaaaagcaggcttc GTA TAC GGC GAC CTT GAC GGT GAC<br>3' ggggaccactttgtacaagaaagctgggtc GTC TTA TTC AAC CGG GAG AGT TTT GCG
ZP_06145446	3	SIGN-CBM22-GH10-CBM22-Doc	5' ggggacaagtttgtacaaaaaagcaggcttc CAG GAA ATG ATC CTG GGT GAC ATC<br>3' ggggaccactttgtacaagaaagctgggtc TTA ATT TGC AGG AAA TTC TCT TAT C
ZP_06144588	4a	UNK-Coh-UNK-Doc (ScaF)	5' ggggacaagtttgtacaaaaaagcaggcttc TTC ACT GAG TAT AAG CTT GGC

			3' ggggaccactttgtacaagaaagctgggtc TTA TGG AGA ATT ATG AGC CTG
ZP_06142016	4a	SIGN-UNK-Doc	5' ggggacaagtttgtacaaaaaagcaggcttc AAC GAG ATG AAC GCC GCA GGA GAC<br>3' ggggaccactttgtacaagaaagctgggtc TCA CAC AGA GCT CTG AGC ATA ATG
ZP_06142361	4a	SIGN-Coh-LNK-Doc (ScaH)	5' ggggacaagtttgtacaaaaaagcaggcttc AAA CCG CAG TAC CGC CTC GGA G<br>3' ggggaccactttgtacaagaaagctgggtc TCA ACC TCT GAG AGG CTG ATG
ZP_06143379	4a	SIGN-Doc-UNK-GH3	5' ggggacaagtttgtacaaaaaagcaggcttc GAG GGA AAT ACC CTC GGC GAC<br>3' ggggaccactttgtacaagaaagctgggtc TCA GAA GGA ATC AGT CAG CCC
ZP_06143695	4a	UNK-LNK-Doc	5' ggggacaagtttgtacaaaaaagcaggcttc GTA AAC ATC AGT TAT ACA TTA GG<br>3' ggggaccactttgtacaagaaagctgggtc TTA AAC ATT TTT GAG TGA ATC
ZP_06144357	4a	SIGN-Doc-UNK	5' ggggacaagtttgtacaaaaaagcaggcttc GAA ACT GAT ATC ATG CAC GGT G<br>3' ggggaccactttgtacaagaaagctgggtc TCA TAT AAC AGT GTC ATT TAC
ZP_06145744	4a	UNK-Coh-Doc (ScaI)	5' ggggacaagtttgtacaaaaaagcaggcttc GCG GTT ATT ATC GGC GAT GTC<br>3' ggggaccactttgtacaagaaagctgggtc TTA TCT GCT TGC GTT TAT AAA TTC
ZP_06145754	4a	SIGN-Doc-UNK	5' ggggacaagtttgtacaaaaaagcaggcttc GCC GGC GGC CAG ACT CAC GGC<br>3' ggggaccactttgtacaagaaagctgggtc TCA AAG GGA TTC AGT GTA GCC
ZP_06142815	4b	SIGN-UNK-X142-UNK-X142-UNK-Doc	5' ggggacaagtttgtacaaaaaagcaggcttc GCC TGC GAG GAC AAA ATG GGG<br>3' ggggaccactttgtacaagaaagctgggtc TTA TTT TCC CTC TGA TGC TGA TGC
ZP_06144573	5	SIGN-X148-LNK-Coh-LNK-Coh-LNK-Doc (ScaA)	5' ggggacaagtttgtacaaaaaagcaggcttc CCT GCA GAA ACA ACA ACT ACA G<br>3' ggggaccactttgtacaagaaagctgggtc TTA GCC CTT AGC AGG GAG TGT GAT G
ZP_06142459	6a	SIGN-X128-LNK-Doc-UNK	5' ggggacaagtttgtacaaaaaagcaggcttc GAT GAA ACT TTC ATC ATG GGT GAC<br>3' ggggaccactttgtacaagaaagctgggtc TCA GTT ATC TGA CAA CAG CAA ACG
ZP_06143476	6a	SIGN-X134-UNK-Doc	5' ggggacaagtttgtacaaaaaagcaggcttc GCA GAC AGT TTC ATT ATG GGT GAC<br>3' ggggaccactttgtacaagaaagctgggtc TTA TTG TTT CAG AAG TTC ACG
ZP_06144432	6a	SIGN-UNK-Doc	5' ggggacaagtttgtacaaaaaagcaggcttc ATA GAT GAT ACA GCT GAC AG<br>3' ggggaccactttgtacaagaaagctgggtc TTA CTG TTT CAG ATA TTC ACG
ZP_06145118	6a	SIGN-UNK-GH18-Doc	5' ggggacaagtttgtacaaaaaagcaggcttc AAG ACT TTC ATT GCA GGC GAT G<br>3' ggggaccactttgtacaagaaagctgggtc TCA TAG CAT TTC CTT TAT AAG
ZP_06142225	6b	SIGN-UNK-PL1-UNK-Doc	5' ggggacaagtttgtacaaaaaagcaggcttc AAC CCG GAT GTT GAG CCT GTT CCG<br>3' ggggaccactttgtacaagaaagctgggtc TTA TTT GCT GAG AGT ATC GAT TAT G
ZP_06142906	6b	SIGN-Doc-UNK	5' ggggacaagtttgtacaaaaaagcaggcttc TCT GCT CTC GAA CCG CCA AGG<br>3' ggggaccactttgtacaagaaagctgggtc TCA ATT TGC GTC AGC AAT GCC
ZP_06143324	6b	SIGN-Doc-UNK	5' ggggacaagtttgtacaaaaaagcaggcttc GAC GCC CCT GCT ATG ACG GGC<br>3' ggggaccactttgtacaagaaagctgggtc TCA ACC ATG AGG GAG CCT GCC
ZP_06144185	6b	SIGN-UNK-Doc	5' ggggacaagtttgtacaaaaaagcaggcttc TGT GAC TGT CAG ATA GGT GAC<br>3' ggggaccactttgtacaagaaagctgggtc TCA TAA AGC AGT TTG GCC TTC
ZP_06142338	-	SIGN-UNK-CBM13-Doc-GH43-UNK-GH43-UNK	5' ggggacaagtttgtacaaaaaagcaggcttc GGA GGC GAG GGT CAG AAA TTC<br>3' ggggaccactttgtacaagaaagctgggtc TCA GCT GTA GTC CTC GCT GTC
ZP_06142740	-	UNK-Doc-UNK	5' ggggacaagtttgtacaaaaaagcaggcttc AAA TAC ACT CCC TCG AAT GTA G<br>3' ggggaccactttgtacaagaaagctgggtc TCA GTC GTA AGC TCC TGT TGA TGC
ZP_06142981	-	SIGN-UNK-X159-X159-X159-X159-X159-X159-X159-X159-UNK-X159-X159-X159-UNK-LNK-Doc-UNK-Doc-UNK	5' ggggacaagtttgtacaaaaaagcaggcttc ACT TCA AAG GAT ACA CTT TAC GGC<br>3' ggggaccactttgtacaagaaagctgggtc TCA TAC GGG GAT AGC AGC CTC GCC
ZP_06144059	-	SIGN-UNK-Doc	5' ggggacaagtttgtacaaaaaagcaggcttc GTC CCC AAA TCA TCA GGC G<br>3' ggggaccactttgtacaagaaagctgggtc TTA GCC GCC GGA GAG CAG
ZP_06145331	-	Sign-GH11-LNK-CBM22-LNK-GH10-LNK-Doc-LNK-GH11-LNK-CE4	5' ggggacaagtttgtacaaaaaagcaggcttc CAG GTT TCT ACA TGG GGC GAT G<br>3' ggggaccactttgtacaagaaagctgggtc TCA CCA CTG ATC ATA TGG CT G

Supplementary Table S5. Cohesin modules of *R. flavefaciens* strain FD-1 selected for the *in vivo* study.

Accession No.	Scaffoldin	Primers used
ZP_06144573	ScaA	5' cacaccatgggagctagc CAG CCT GTT GCT AAT GCA GAC<br>3' cacactcgagtta TGG ATC ATC AAC AGG GTT ACC
ZP_06144574	ScaB	5' cacaccatgggagctagc CCT GTA GCT AAC GCT GAT G<br>3' cacactcgagtta GCC CTC CTC ATT AGG AGT ACC
ZP_06144574	ScaB	5' cacaccatgggagctagc aag aat gta aca cct gct aca g<br>3' cacactcgagtta AAC TAC AGG TGT ATC ACC AAC
ZP_06144574	ScaB	5' cacaccatgggagctagc GCT AAG GGT TCA GTA AAA TGG<br>3' cacactcgagtta TGA ATC AGG AGT CTT AAC
ZP_06144572	ScaC	5' cacaccatgggagctagc GCA GGC GAA ACA GTG<br>3' cacactcgagtta TAC TTC TGC TGA AGG AAC
ZP_06144576	ScaE	5' cacaccatgggagctagc CTC ACA GAC AGA GGA ATG<br>3' cacactcgagtta CTC AGG CTC ACC AGC CTT GAT TG
ZP_06142991	ScaG	5' cacaccatgggagctagc GCT GAC GGC GGT TTC ACA GAC<br>3' cacactcgagtta GAT ATA GCC GTC CTT CAT GCC
ZP_06142361	ScaH	5' cacaccatgggagctagc GCC TGC CCA GAT CGT GGA AAC<br>3' cacactcgagtta TTC GGA AGG AGC GGT TAT CTC
ZP_06144588	ScaF	5' cacaccatgggagctagc aat tca aca gat ctc acc<br>3' cacactcgagtta TTT TTT CTC GCC GAG TAT CCT G
ZP_06145744	ScaI	5' cacaccatgggagctagc AAG CCT GTG CTG CGC ATC<br>3' cacactcgagtta CGA GAA AAT GTG CTT GTT CAT TG

Supplementary Table S6. Set of primers used to generate G10A/R11A and G48A/R49A mutations in the XynDoc constructs of peptidase-Doc (ZP_06142181) and ScaH-Doc (ZP_06142361) for testing the dual-binding mode in these type III dockerins. Red fonts designate restriction binding sites and blue fonts the substituted nucleic bases on the mutagenic primers.

		**ZP_06142181**	**ZP_06142361**
KpnI site	F[1]	GCTAC**GGTACC**TCTCACACTGCTTCTGAAACGT	GCTAC**GGTACC**TAAACCGCAGTACCGCCTC
BamHI site	R[4]	CGCCAG**GGATCC**TTACTAATTTATTACAGATGATTTAGC	CGCCAG**GGATCC**TTATCAACCTCTGAGAGGCTG
G10A R11A	F[2]	GAACGGAATAGTAGACG**CCGC**AGATGCTACACTGGTGC	CAACGGAATTATTGAC**GCAGCT**GATGCGACCGCAGTCC
	R[3]	GCACCAGTGTAGCATCT**GCGG**CGTCTACTATTCCGTTC	GGACTGCGGTCGCATC**AGCTGC**GTCAATAATTCCGTTG
G48A R49A	F[2]	CAGCAATGACATCATCGACG**CAGC**AGATGCTACAGAAATACTTAC	GGATAACATGATAGAC**GCAGCT**GACGCTACACATATCC
	R[3]	GTAAGTATTTCTGTAGCATCT**GCTG**CGTCGATGATGTCATTGCTG	GGATATGTGTAGCGTC**AGCTGC**GTCTATCATGTTATCC

Supplementary Figures

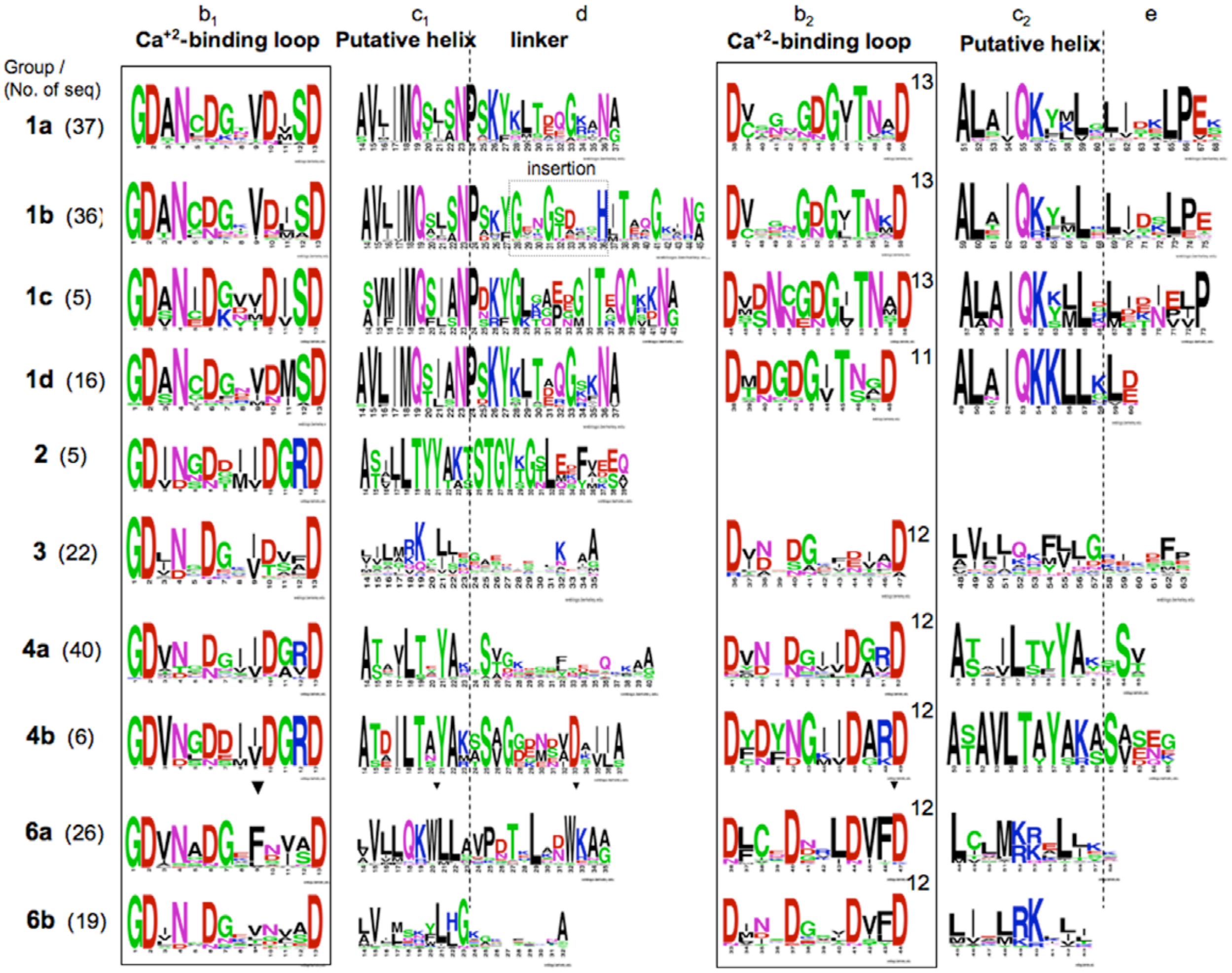

Figure S1. Conservation patterns of different dockerin groups from *R. flavefaciens* FD-1. The 222 dockerins were clustered into groups by Rincon et al[33] according to their conserved sequence features, and their sequence logo is presented. The length of the second repeat is marked for each group.

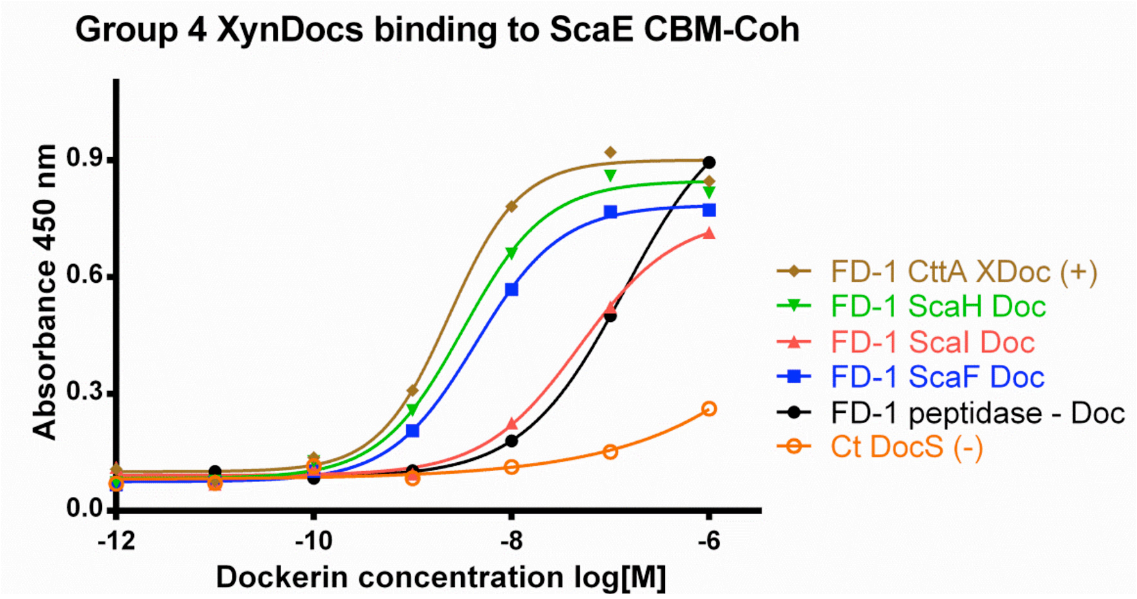

Figure S2. Binding of group-4 dockerins to ScaE cohesin probed by an ELISA assay. XynDocs of CttA XDoc, ScaH, ScaF, peptidase-Doc and ScaI were purified on Ni-NTA columns and interacted with the ScaE cohesin. *C. thermocellum* DocS was chosen as a negative control. From the IC_{50} values it is clear that these dockerins indeed bind the ScaE cohesin, even though only weak interaction was observed using the cellulose microarray approach. Surprisingly, the interaction is relatively strong when compared with other known protein-protein interactions.

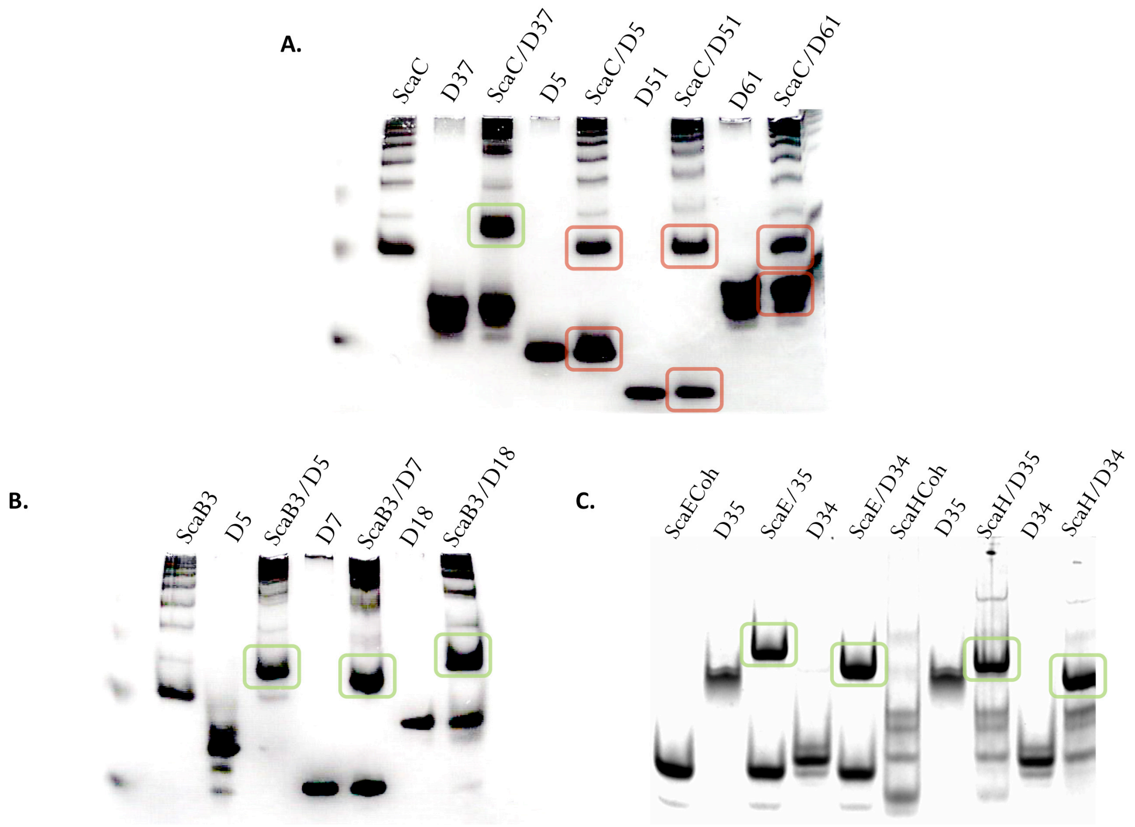

Figure S3. Confirmation of *in vivo* co-expression data by non-denaturing PAGE. The first lane of gels A and B were loaded with the cohesin (ScaC in A and ScaB3 in B). Adjacent lanes were loaded with a test dockerin and with both cohesin and dockerin modules together after 60-min incubation at equimolar concentrations. Dockerins are numbered according to Table 1. The appearance of a band with a different migration pattern (green highlights) in lanes containing the complex represents a positive result (e.g. ScaC/D37), while a negative result (e.g. ScaC/D5) is given by the appearance of only the individual dockerin and cohesin bands (red highlights). ScaC interacts with group-3 dockerin D37 but not with groups-1, -4 or -6 dockerins D5, D51 or D61. ScaB3 binds to group-1 dockerins D5, D7 and D18. Gel C shows the binding of both group-2 dockerins to ScaE and ScaH.

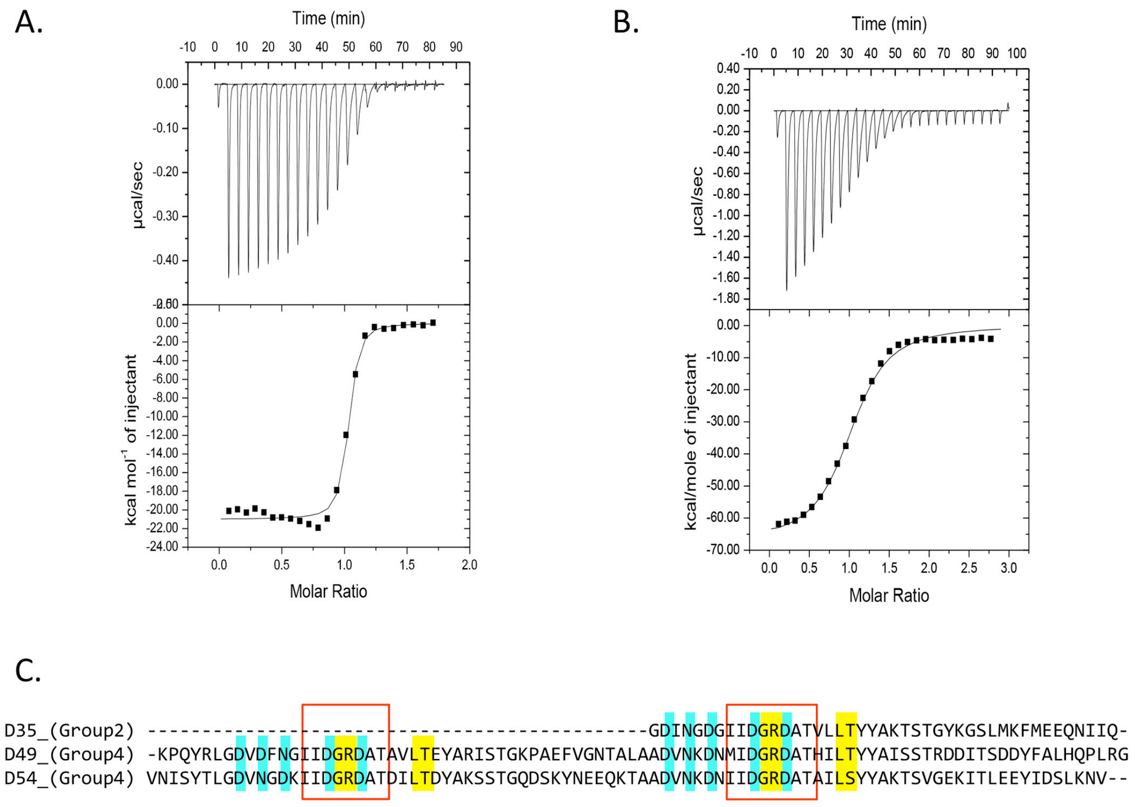

Figure S4. Binding of group-2 and group-4 dockerins to ScaE evaluated by ITC. The dockerins are numbered according to Table 1. Representative titrations are displayed in panel (A), ScaE Coh and dockerin 35 (D35), and panel (B), ScaE Coh and dockerin 49 (D49). The upper part of each panel shows the raw heats of binding, whereas the lower parts comprise the integrated heats after correction for heat dilution. The curve represents the best fit to a single-site binding model. (C) Alignment of dockerin D35 (group 2) with two group-4 dockerins, D49 (ScaHDoc) and D54. The conservation of the postulated cohesin recognition site is highlighted with a red box. Residues involved in Ca^{+2}-binding are colored in cyan while putative residues involved in cohesin recognition are highlighted in yellow.

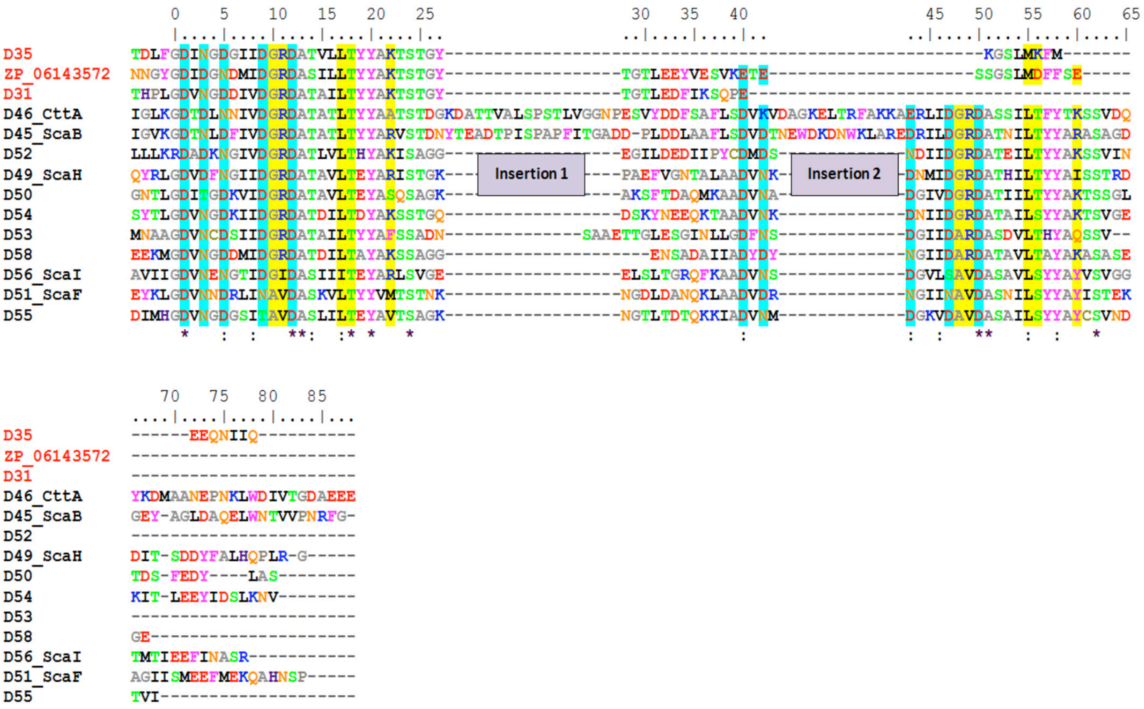

Figure S5. Alignment of the dockerins belonging to groups 4 and 2. Group-4 dockerins exhibit an atypical two-fold symmetry that resembles modules of type I rather than type III, prevalent in *R. flavefaciens*. The dominant cohesin-recognition residues at positions 10-11 and 17-18 of the two repeats are **Gly**/Ala-**Arg**/Ile/Val and **Leu-Thr**/Ser, respectively. Interestingly, the three dockerins of group-2 (marked in red), comprising the first Ca^{+2}-binding loop-helix motif alone, are remarkably conserved with respect to the canonical 1st helix-loop segment of group-4 dockerins. The alignment was performed in Clustal Omega (http://www.ebi.ac.uk/Tools/msa/clustalo/). Dockerins are numbered according to Table 1. Note the insertions of group-4 ScaB and CttA XDocs that are absent in other members of the group. These insertions have been shown to form supporting buttresses that interact with the upstream X-module of the above-mentioned scaffoldins (23,30). Residues involved in Ca^{+2}-binding are designated in cyan while residues involved in cohesin recognition are highlighted in yellow.
